# Genetically determined hypoalbuminemia as a risk factor for hypertension: instrumental variable analysis

**DOI:** 10.1038/s41598-021-89775-3

**Published:** 2021-05-28

**Authors:** Jong Wook Choi, Joon-Sung Park, Chang Hwa Lee

**Affiliations:** 1 Research Institute of Medical Science, Konkuk University School of Medicine, Chungju, South Korea; 2 Department of Internal Medicine, Hanyang University College of Medicine, 222 Wangsimni-ro, Seongdong-gu, Seoul, 04763 South Korea

**Keywords:** Epidemiology, Genetics research, Hypertension

## Abstract

Hypoalbuminemia is associated with vascular endothelial dysfunction and the development of chronic cardiovascular diseases. However, the relationship between serum albumin concentration and blood pressure changes remains controversial. Community-based longitudinal cohort data collected from Korean Genome and Epidemiology Study were used in this study. Hypoalbuminemia was defined as a serum albumin concentration of ≤ 4.0 g/dL. A total of 4325 participants were categorized into control (n = 3157) and hypoalbuminemia (n = 1168) groups. Serum albumin had a non-linear relationship with the risk of hypertension development. A genome-wide association study revealed 71 susceptibility loci associated with hypoalbuminemia. Among susceptibility loci, genetic variations at rs2894536 in *LOC107986598* and rs10972486 in *ATP8B5P* were related to elevated blood pressure. Serum albumin (HR = 0.654, 95% CI 0.521–0.820) and polymorphisms of rs2894536 (HR = 1.176, 95% CI 1.015–1.361) and rs10972486 (HR = 1.152, 95% CI 1.009–1.316) were significant predictors of hypertension development. Increased albumin concentration instrumented by 2 hypoalbuminemia-associated SNPs (rs2894536 and rs10972486) was associated with decreased HRs for hypertension development (HR = 0.762, 95% CI 0.659–0.882 and HR = 0.759, 95% CI 0.656–0.878). Our study demonstrated that genetically determined hypoalbuminemia is a significant predictor of incipient hypertension.

## Introduction

Albumin, one of the major serum proteins, has multiple important physiological functions involving stabilization of plasma colloid osmotic pressure, transportation of diverse substances, and significant antioxidant activity, and its concentration is finely regulated by various systems in the physiologic state^1^. Hypoalbuminemia is strongly related to unfavorable health outcomes in various pathologic conditions, including hospitalized patients, surgical patients, and those with heart failure, chronic liver disease, chronic kidney disease, or end-stage renal disease^2–4^. Furthermore, recent epidemiologic studies have shown that low serum albumin concentration is a reliable clinical biomarker of vascular endothelial dysfunction and is an important predictor of future cardiovascular diseases (CVDs) and all-cause mortality in the general population^5–8^.

Hypertension is a well-known modifiable risk factor in the development and progression of chronic CVDs^9^. The vascular endothelium is the primary site of systemic hemodynamic dysfunction in various metabolic and vascular diseases^10^. Vascular endothelial dysfunction is mainly characterized by the induction of a pro-inflammatory or pro-thrombotic state and impairment in endothelium-dependent relaxation of blood vessels^10,11^, which may play critical roles in the pathogenesis of hypertension. However, the pathophysiologic connection between hypoalbuminemia and hypertension has not yet been clarified.

There has been inconsistent evidence on the relationship between serum albumin concentration and the risk of hypertension. Previously, Hu et al. and Høstmark et al. showed that a rise in serum albumin levels was associated with elevated systolic blood pressure (SBP) and diastolic blood pressure (DBP) in a Caucasian population^12,13^. Recently, Oda et al. demonstrated that a decreased serum albumin level was also a significant predictor of hypertension in a Japanese health population^14^. Such findings may indicate that possible confounders, such as differences across racial groups and selection bias, could alter the impact of serum albumin levels on the development of hypertension. Instrumental variables can be used to help address the effect of unobserved confounders by operating as a randomization process when evaluating the association between environmental exposures and outcomes of interest. Thus, we performed a genome-wide association study (GWAS) to identify genetic variants associated with hypoalbuminemia and instrumental variable analysis to establish robust causal relationships between genetically determined hypoalbuminemia and the development of hypertension in a community-based cohort population.

## Results

### Baseline characteristics

The participants (n = 4325) comprised 1959 men and 2366 women, with a mean age of 49.5 ± 8.2 years. They were categorized into two groups according to their serum albumin levels. Participants with hypoalbuminemia were older and more likely to have elevated DBP, decreased hemoglobin, increased eGFR, and poor lipid profiles; there were more female subjects than male subjects with hypoalbuminemia. Participants with normal serum albumin levels had increased white blood counts, fasting glucose, and postprandial glucose. Other demographic data and clinical characteristics are presented in Table 1.

**Table 1:** Baseline characteristics grouped by serum albumin concentration.

Variable	Albumin (g/dL)		P
	> 4	≤ 4	
	(n = 3157)	(n = 1168)	
Age (year)	48.9 ± 7.9	51.1 ± 8.7	< 0.0001
Sex (male, %)	1625 (51.5)	334 (28.6)	< 0.0001
Smoker (n, %)	889 (28.2)	226 (19.3)	< 0.0001
Body mass index (kg/m^2^)	24.0 ± 3.1	23.9 ± 2.9	0.6863
Waist circumference (cm)	80.5 ± 8.6	79.9 ± 8.2	0.1709
Systolic BP (mmHg)	113.4 ± 10.8	113.2 ± 11.1	0.4836
Diastolic BP (mmHg)	76.0 ± 7.3	74.7 ± 7.5	< 0.0001
White blood cell (10^9^/L)	6.4 ± 1.7	6.3 ± 1.9	0.0113
Hemoglobin (g/dL)	13.6 ± 1.5	12.8 ± 1.5	< 0.0001
Platelet (10^3^/μL)	263.1 ± 61.5	259.9 ± 61.7	0.5100
Total protein (g/dL)	7.4 ± 0.4	6.9 ± 0.3	< 0.0001
Albumin (g/dL)	4.4 ± 0.3	3.9 ± 0.1	< 0.0001
Calcium (mg/dL)	9.3 ± 0.6	9.5 ± 0.4	< 0.0001
Fasting glucose (mg/dL)	82.9 ± 8.4	79.5 ± 6.9	< 0.0001
Post-prandial glucose (mg/dL)	113.8 ± 29.9	109.3 ± 27.9	0.0001
Hemoglobin A1c (%)	5.50 ± 0.34	5.51 ± 0.34	0.3316
eGFR^a^ (mL/min/1.73 m^2^)	93.2 ± 13.7	97.7 ± 11.3	< 0.0001
Total bilirubin (mg/dL)	0.7 ± 0.3	0.5 ± 0.2	< 0.0001
Aspartate aminotransferase (IU/L)	28.2 ± 13.5	28.5 ± 19.4	< 0.0001
Alanine aminotransferase (IU/L)	26.2 ± 20.6	24.3 ± 22.8	< 0.0001
γ-Glutamyl transferase (IU/L)	28.3 ± 34.4	26.1 ± 71.9	< 0.0001
Triglyceride (mg/dL)	135.9 ± 86.0	146.0 ± 90.1	< 0.0001
HDL-cholesterol (mg/dL)	45.7 ± 10.1	44.0 ± 9.7	< 0.0001
LDL-cholesterol (mg/dL)	101.5 ± 27.8	118.0 ± 31.2	< 0.0001
C-reactive protein (mg/dL)	0.19 ± 0.39	0.23 ± 0.55	0.2859
UACR (mg/g Cr)	10.1 ± 6.2	9.7 ± 6.7	0.3833

Results are expressed as the mean ± standard deviation or as frequencies (and proportions).

BP, blood pressure; eGFR, estimated glomerular filtration rate; HDL, high-density lipoprotein; LDL, low-density lipoprotein; UACR, urine albumin/creatinine ratio; Cr, creatinine.

^a^Estimated using the Chronic Kidney Disease Epidemiology Collaboration equation.

### Relationship between hypoalbuminemia and blood pressure

**Figure 1:**
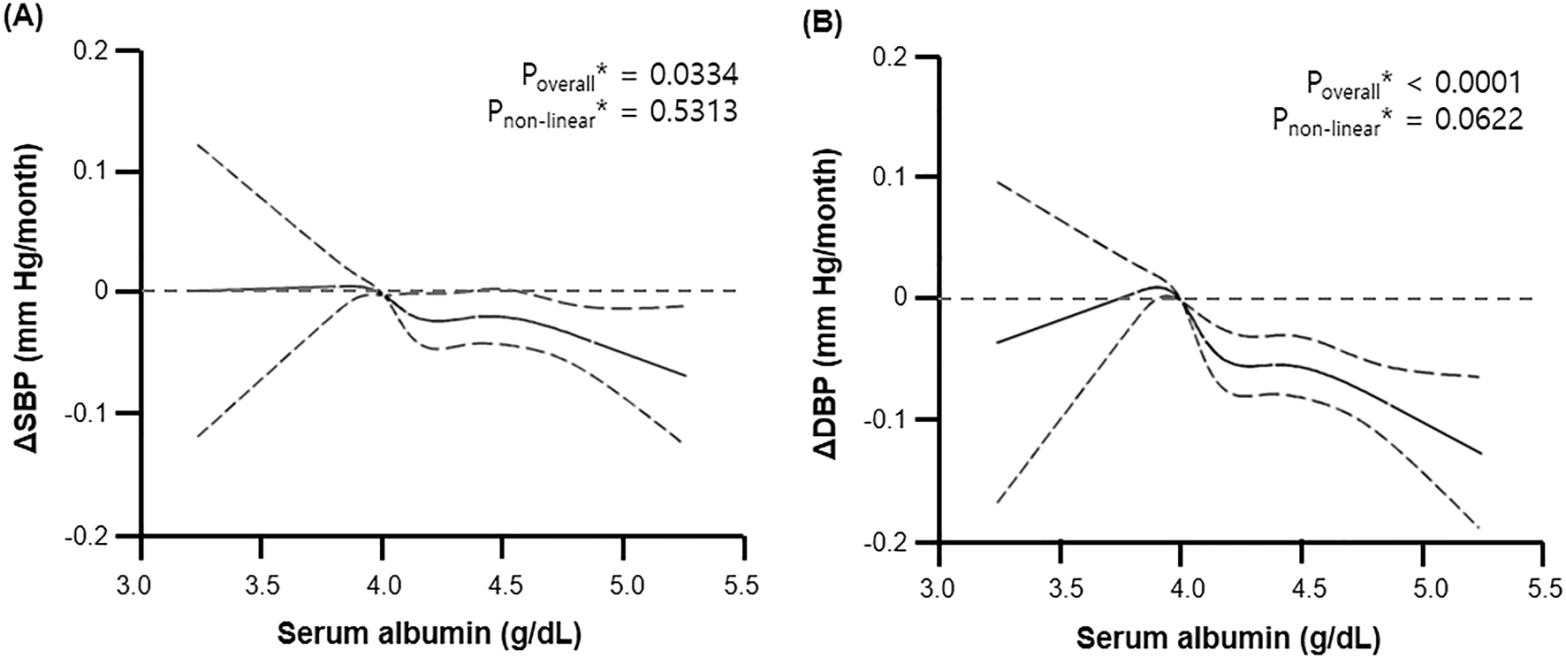
Relationship of serum albumin levels with the change in (**A**) systolic blood pressure (ΔSBP) and (B) diastolic blood pressure (ΔDBP) compared with the chosen reference albumin (g/dL) of 4.0. The solid line represents the change in BP during the follow-up period, and dashed lines represent 95% confidential intervals. *Calculated by restricted cubic spline regression using age, sex, and smoking history as covariates.

We performed linear regression analysis using age, sex, and smoking history as covariates and found that serum albumin levels were closely related with DBP, hemoglobin, platelet, total protein, calcium, fasting glucose, postprandial glucose, eGFR, total bilirubin, γ-glutamyl transferase, HDL-cholesterol, LDL-cholesterol, and C-reactive protein levels (Table 2). However, our restricted cubic spline regression analysis showed that there may be a non-linear relationship between serum albumin concentration and changes in blood pressure (Fig. 1).

**Table 2:** Multiple linear regression analysis for serum albumin.

Parameter	Crude		Model I	
	*β*	P	*β*	P
Age (year)	− 0.0070	< 0.0001		
Sex (vs male)	0.2021	< 0.0001		
Smoking history (vs never smoker)	− 0.0707	< 0.0001		
Systolic BP (mmHg)	− 0.0001	0.9211		
Diastolic BP (mmHg)	− 0.0047	< 0.0001	− 0.0005	< 0.0001
Body mass index (kg/m^2^)	− 0.0031	0.4377		
Waist circumference (cm)	− 0.0005	0.4193		
White blood cell (10^9^/L)	0.0104	0.0002	0.0029	0.2720
Hemoglobin (g/dL)	0.0647	< 0.0001	0.0391	< 0.0001
Platelet (10^3^/μL)	0.0006	0.0006	0.0004	< 0.0001
Total protein (g/dL)	0.5119	< 0.0001	0.4824	< 0.0001
Calcium (mg/dL)	− 0.2856	< 0.0001	− 0.2686	< 0.0001
Fasting glucose (mg/dL)	0.0141	< 0.0001	0.0117	< 0.0001
Post-prandial glucose (mg/dL)	0.0011	< 0.0001	0.0016	< 0.0001
Hemoglobin A1c (%)	− 0.0284	0.0517		
eGFR (mL/min/1.73 m^2^)	− 0.0093	< 0.0001	− 0.0077	< 0.0001
Total bilirubin (mg/dL)	0.3306	< 0.0001	0.2403	< 0.0001
Aspartate aminotransferase (IU/L)	− 0.0001	0.8388		
Alanine aminotransferase (IU/L)	0.0008	0.0006	0.0003	0.1320
γ-Glutamyl transferase (IU/L)	0.0003	0.0001	0.0002	0.0219
Triglyceride (mg/dL)	− 0.0002	0.0001	− 0.0001	0.5488
HDL-cholesterol (mg/dL)	0.0051	< 0.0001	0.0037	< 0.0001
LDL-cholesterol (mg/dL)	− 0.0037	< 0.0001	− 0.0037	< 0.0001
C-reactive protein (mg/dL)	− 0.0385	0.0002	− 0.0300	0.0075
UACR (mg/g Cr)	0.0008	0.2875		

Model I: adjusted for age, sex, and smoking history.

### De novo GWAS for hypoalbuminemia

We examined genetic data from 1168 participants with hypoalbuminemia (case) and 3157 participants with non-hypoalbuminemia (control) from the Ansan-Anseong cohort of the Korean Genome and Epidemiology Study (KoGES). After a standard quality control procedure, we obtained genotyping results for 519,364 SNPs and generated a quantile–quantile plot (Supplementary Fig. S1). The genomic inflation factor λ was 1.0206 in the quantile-quantile plot and the observed p-values show an early deviation from expectation, which may be indicative of the inflation in test statistics caused by polygenic effect and Korean population structure.

We performed a logistic regression analysis for slightly decreased serum albumin concentration, using age and sex as covariates, and subsequently calculated the minimum P-value for three genetic models (additive, recessive, and dominant, Supplementary Table S1). Our association analysis revealed that a total of 71 SNPs from 28 distinct genomic regions were significantly associated with hypoalbuminemia (P_GWAS_ ranging from 9.9 × 10^–5^ to 6.5 × 10^–6^, Supplementary Fig. S2). Among the loci found in this study, only one genetic locus, *GCKR,* was revealed in other GWAS of populations of eastern Asian ancestry^15^.

Because the SNPs of interest were located in a genomic region encoding *VEGFA, LOC107986598, LOC100132354, UNC13B, ATP8B5P,* and *RUSC2*, among others, we performed imputation analysis to characterize these loci. The regional association plots using genotyped and imputed data showed that rs2894536 was confined to regions around the *LOC107986598* gene and rs10972486, the *ATP8B5P* gene (Supplementary Fig. S3).

### Hypoalbuminemia, hypoalbuminemia-related SNPs, and the risk of hypertension development

To avoid bias from the endogeneity in conventional linear regression, we performed two-stage least squares estimation analysis using genetic variant(s) explaining the change in serum albumin concentration as instrument variables, including rs2894536 in *LOC107986598* and rs10972486 in *ATP8B5P* (Table 3 and Supplementary Table S2). In ordinary least square linear regression, SBP and DBP decreased with increasing serum albumin levels (ΔSBP, β = − 0.043 mmHg/month, P = 0.0017; ΔDBP, β = − 0.103 mmHg/month, P < 0.0001). Our two-stage least squares estimation analysis revealed a possible causal role of hypoalbuminemia in determining SBP change (rs2894536: F = 16.56, P_DWH_ = 0.0009; rs10972486: F = 16.69, P_DWH_ = 0.0008), but not DBP.

**Table 3:** Relationship of serum albumin (endogenous variable) with the change in (A) systolic blood pressure (ΔSBP) and diastolic blood pressure (ΔDBP) as tested both by ordinary least squares linear regression and the application of two-stage least squares regression analysis using candidate genetic polymorphisms as an instrument variable.

Instrumental variable	ΔSBP (mmHg/month)					ΔDBP (mmHg/month)							
	β	SE	P	F	P_DWH_^a^	β	SE		P		F		P_DWH_^a^
**Ordinary least square linear regression**													
Albumin (g/dL)	− 0.043	0.014	0.0017			− 0.103		0.017		< 0.0001			
**Two-stage least squares regression analysis**													
rs2894536 (vs. GG)	− 0.050	0.007	< 0.0001	16.56	0.0009	0.001		0.008		0.9921		5.06	0.1675
rs10972486 (vs. TT)	− 0.051	0.007	< 0.0001	16.69	0.0008	− 0.001		0.008		0.9043		4.97	0.1737

Regression results were adjusted for age and sex.

SE, standard error.

^a^Estimated using the Durbin-Wu-Hausman test, which examines the difference between the estimates from ordinary least squares linear regression and instrumental variable analysis.

**Figure 2:**
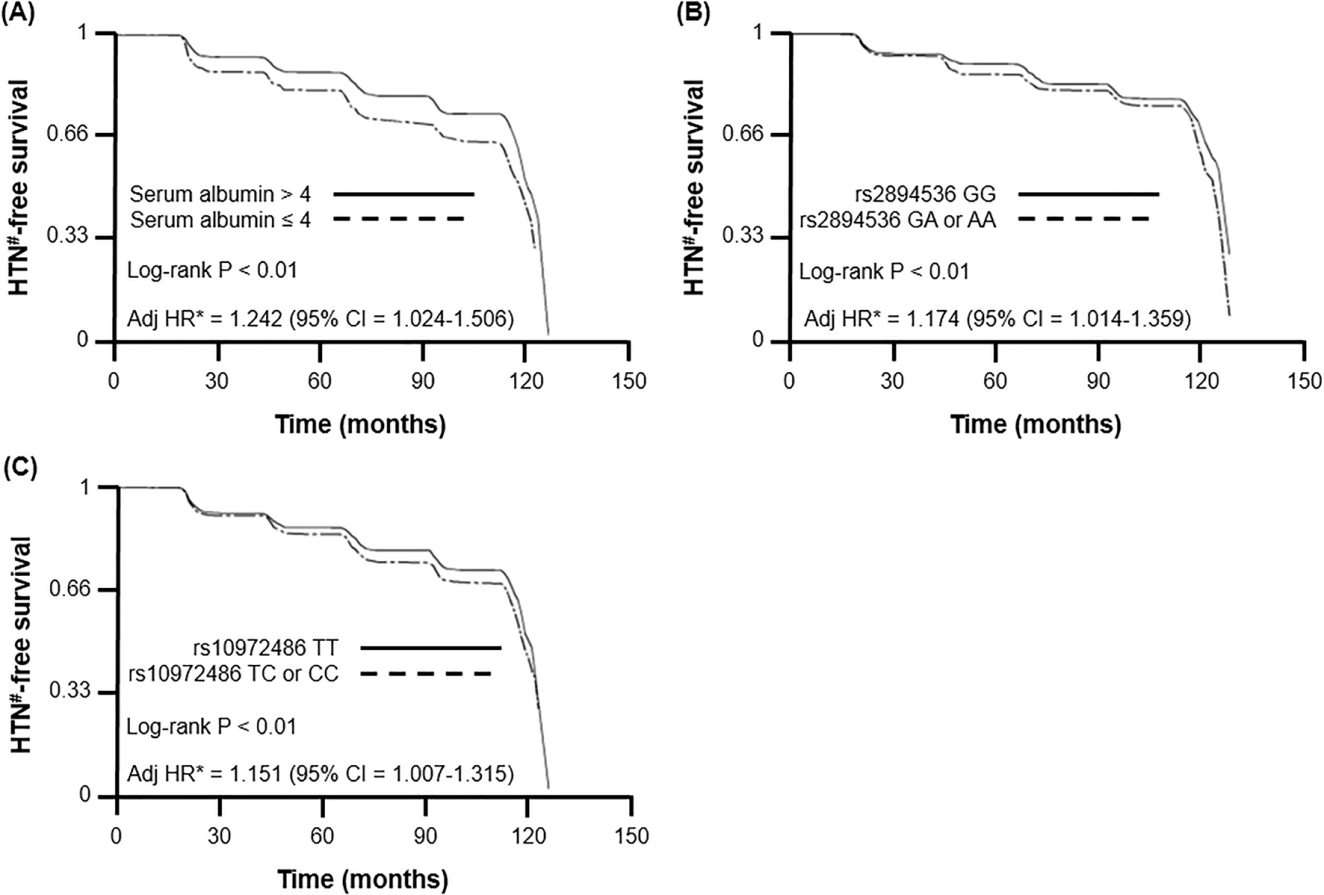
Hypertension (HTN)^#^-free survival curves according to the multiple Cox-proportional hazards model for (**A**) serum albumin, (**B**) polymorphism at rs2894536 in LOC107986598, and (**C**) polymorphism at rs10972486 in ATP8B5P. *Adjusted for age, sex, smoking history, systolic BP, diastolic BP, body mass index, waist circumference, hemoglobin, platelet, albumin, hemoglobin A1c, alanine aminotransferase, γ-glutamyl transferase, triglyceride, and HDL-cholesterol levels. ^**#**^Defined as BP ≥ 140/90 mmHg and/or antihypertensive drug therapy during the follow-up period.

Our multiple Cox-proportional hazards model demonstrated that serum albumin concentration and hypoalbuminemia-related SNPs were deeply associated with an increased risk of hypertension development (albumin, HR = 0.654, 95% CI 0.521–0.820; rs2894536, HR = 1.176, 95% CI 1.015–1.361; rs10972486, HR = 1.152, 95% CI 1.009–1.316) after adjustment for age, sex, smoking history, SBP, DBP, body mass index, waist circumference, hemoglobin, platelet, hemoglobin A1c, alanine aminotransferase, γ-glutamyl transferase, TG, and HDL-cholesterol level (Table 4 and Supplementary Table S3). Subsequent survival analyses with multiple Cox-proportional hazards regression analysis and log-rank test were performed to compare hypertension-free survival between the groups. We found that participants with serum albumin ≤ 4.0 g/dL and polymorphisms of rs2894536 or rs10972486 had poor hypertension-free survival rate (Fig. 2).

**Table 4:** Multiple Cox proportional hazard model for hypertension (HTN)^a^ development.

Variable	Model I		Model II		Model III	
	HR	95% CI	HR	95% CI	HR	95% CI
Albumin (g/dL)	0.599	0.484–0.741	0.654	0.521–0.820		
**rs2894536**						
Additive model	1.164	1.022–1.325	1.158	1.017–1.319	1.141	1.003–1.299
Dominant model	1.188	1.026–1.376	1.185	1.024–1.371	1.176	1.015–1.361
Recessive model	1.225	0.777–1.931				
**rs10972486**						
Additive model	1.090	0.990–1.201				
Dominant model	1.160	1.015–1.325	1.153	1.008–1.317	1.152	1.009–1.316
Recessive model	1.047	0.854–1.282				

Model I: adjusted for age, sex, and smoking history.

Model II: adjusted for age, sex, smoking history, systolic BP, diastolic BP, body mass index, waist circumference, hemoglobin, platelet, hemoglobin A1c, alanine aminotransferase, γ-glutamyl transferase, triglyceride, and HDL-cholesterol levels.

Model III: adjusted for age, sex, smoking history, systolic BP, diastolic BP, body mass index, waist circumference, hemoglobin, platelet, albumin, hemoglobin A1c, alanine aminotransferase, γ-glutamyl transferase, triglyceride, and HDL-cholesterol levels.

HR, hazard ratio; *CI*, confidence interval.

^a^Defined as BP ≥ 140/90 mmHg and/or antihypertensive drug therapy during the follow-up period.

**Figure 3:**
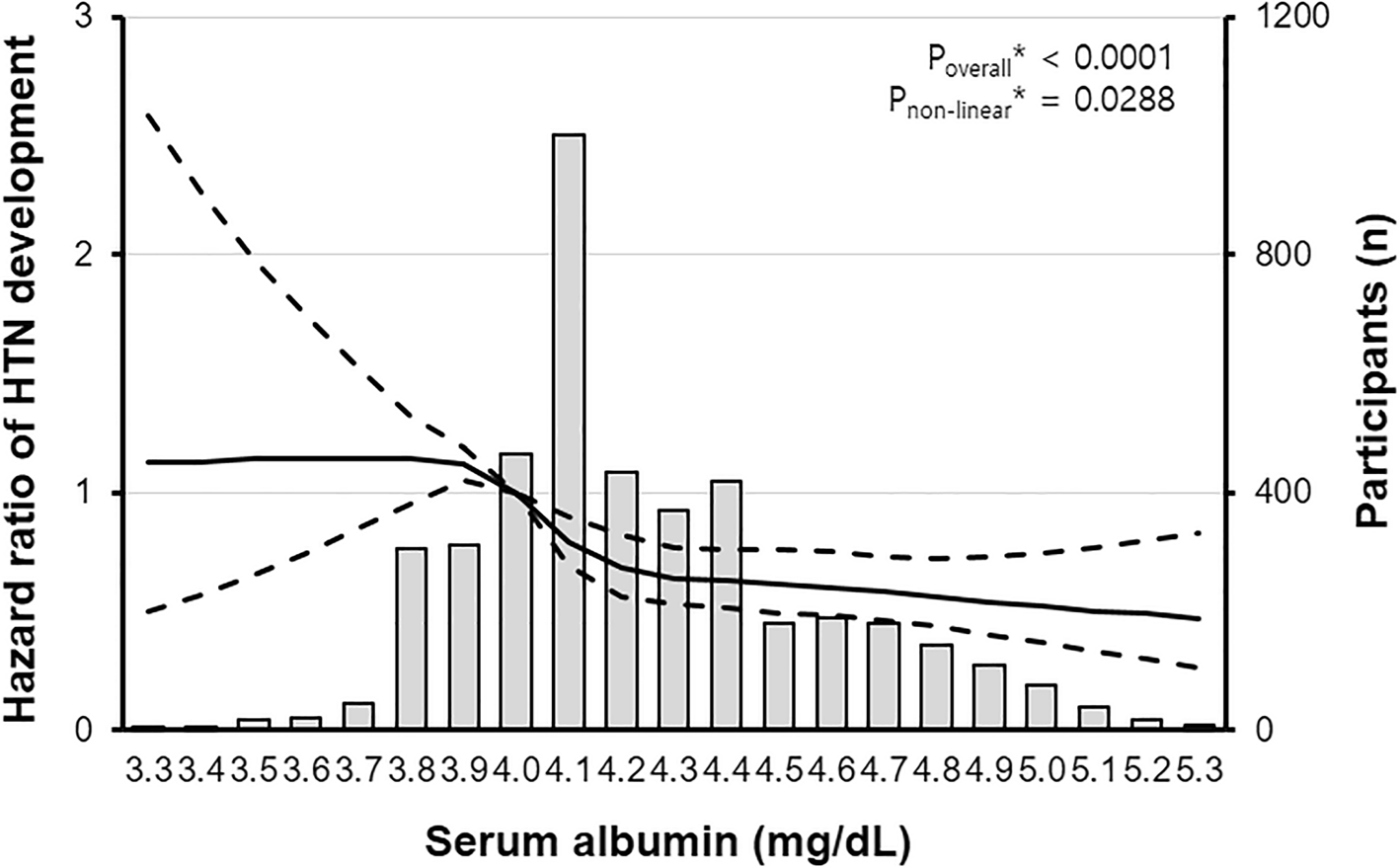
Non-linear relationship between serum albumin levels and the hazard ratio of hypertension development compared with the chosen reference albumin (g/dL) of 4.0. The solid line represents the hazard ratio of hypertension during the follow-up period, and dashed lines represent 95% confidential intervals. *Calculated using restricted cubic spline regression using age, sex, smoking history, systolic BP, diastolic BP, body mass index, waist circumference, hemoglobin, platelet, hemoglobin A1c, alanine aminotransferase, γ-glutamyl transferase, triglyceride, and HDL-cholesterol level as covariates.

We performed restricted cubic spline regression analysis to evaluate the linearity of the relationship between serum albumin concentration and the risk of hypertension development and found that there was a non-linear relationship between them (Fig. 3). The inverse relation of serum albumin with the risk of hypertension development was attenuated in the condition of low serum albumin level (below 4 g/dL), suggesting that other confounding factor(s) may change the effect of serum albumin level on arterial blood pressure. To draw causal inferences about the effects of candidate genetic variation on hypoalbuminemia-related hypertension while controlling for unobserved confounding effects, we performed a two-stage residual inclusion analysis and found that genetically determined hypoalbuminemia was deeply related to an increased risk of incident hypertension (rs2894536, HR = 0.762, 95% CI 0.659–0.882; rs10972486, HR = 0.759, 95% CI 0.656–0.878; Table 5).

**Table 5:** Control function instrument variable estimator of the casual HR for the effect of serum albumin levels on HTN development.

Model	Albumin (g/dL)	
	HR^a^	95% CI^a^
**Dominant model of *LOC107986598* polymorphism at rs2894536**		
Adjusted for age, sex, and smoking history	0.727	0.632–0.836
Adjusted for age, sex, smoking history, systolic BP, diastolic BP, body mass index, waist circumference, hemoglobin, platelet, hemoglobin A1c, alanine aminotransferase, γ-glutamyl transferase, triglyceride, and HDL-cholesterol level	0.762	0.659–0.882
**Dominant model of *ATP8B5P* polymorphism at rs10972486**		
Adjusted for age, sex, and smoking history	0.723	0.629–0.831
Adjusted for age, sex, smoking history, systolic BP, diastolic BP, body mass index, waist circumference, hemoglobin, platelet, hemoglobin A1c, alanine aminotransferase, γ-glutamyl transferase, triglyceride, and HDL-cholesterol level	0.759	0.656–0.878

^a^Calculated using a two-stage residual inclusion method.

## Discussion

This study provides comprehensive information on the association between genetic variations, hypoalbuminemia, and the risk of hypertension, showing that genetically determined hypoalbuminemia played a causal role in the development of incipient hypertension. To the best of our knowledge, this is the first instrumental variable study to demonstrate that the vascular protective effect of serum albumin may attenuate the risk of hypertension development.

In this study, we found that there was a reverse relationship between serum albumin and the risk of incipient hypertension. Previous epidemiologic and experimental studies demonstrated that serum albumin with anti-inflammatory and antioxidant properties plays homeostatic regulatory roles in various physiological processes, and hypoalbuminemia is strongly associated with systemic inflammatory responses, vascular endothelial injury, and development of chronic vascular diseases^16–19^. However, other clinical studies showed very inconsistent association between serum albumin level and arterial blood pressure^12–14^. Such findings seem to be the result of not only the limitations of study design, problems of small sample size, or differences in race and ethnicity, but also the hidden effects of confounding variable(s). To overcome the limitations of previous studies and to assess more robust associations between exposure variables and outcome variables, we performed an instrumental variable analysis based on a community-based longitudinal study with a large sample size and demonstrated that a low serum albumin concentration could be a factor for predicting the development of hypertension in the general population.

Our study results revealed that potential genetic variations could be related to changes in the function of vascular endothelial cells and the development of hypertension, showing that two candidate loci, rs2894536 in the *LOC107986598* region and rs10972486 in the *ATP8B5P* region, were deeply associated with hypoalbuminemia. *LOC107986598* is an uncharacterized non-coding ribonucleic acid (ncRNA) gene located near vascular endothelial growth factor A (VEGF-A). Previous basic research demonstrated that ncRNA can exert more widespread effects on gene regulation; VEGF-A is one of the critical molecules for endothelial maintenance and exerts pleiotropic actions to facilitate migration, proliferation, and survival of vascular endothelial cells; and anti-VEGF-A therapies are strongly related to the development of proteinuria and increased blood pressure^20–22^. Such findings suggested that the regulatory function of *LOC107986598* may be important for maintaining physiological vascular endothelial function. Meanwhile, there is limited evidence supporting the relationship between *ATP8B5P* and hypoalbuminemia. *ATP8B5P*, a human gene related to *ATP8B1*, is a typical pseudogene predominantly expressed in the testes. Although the transcript of *ATP8B5P* does not contain fully functional reading frames, several recent experimental studies demonstrated that the ATP8B1 protein belongs to the family IV of P-type adenosine triphosphatase, which is one of the membrane proteins that are responsible for the generation and maintenance of phospholipid asymmetry in the lipid bilayer; ATP8B1 protein was enriched in cerebral micro-vessels; and some pseudogenes appear to harbor the potential to regulate their protein-coding cousins^23–26^. These findings suggest that the VEGFA-or ATP8B1-related pathways may play a role in the development of hypoalbuminemia and initiation of blood pressure elevation. There is a need to further investigate the specific gene regulatory pathway, which will provide target genes for future drug development endeavors.

There was a difference between conventional linear regression analysis and two-stage least squares estimation analysis results: serum albumin level was strongly related with only DBP in conventional linear regression analysis, but it was related to both SBP and DBP in the two-stage least squares estimation analysis. A possible explanation for this discrepancy may be the limitations of observational epidemiology (confounding, reverse causality, or regression dilution bias) for making causal inferences^27^. These findings indicated that caution is needed when drawing conclusions from conventional observational epidemiologic studies, and instrumental variable methods can make more accurate causal relationships between the exposure to the candidate risk factors and clinical outcomes.

There are some limitations to this study. First, statistical methods of causal inference, namely instrumental variable analyses, could not completely remove confounding and selection bias. To avoid a possible current limitation of the instrumental variable analysis, we had strict inclusion criteria and extensive exclusion criteria. Second, either the determination of serum albumin concentration or the development of hypertension might be influenced by a wide variety of factors promoting vascular endothelial dysfunction. Because this community-based cohort study did not contain data on 24-h urine collection, ambulatory BP monitoring, vascular flowmetry, thyroid function test, abdominal ultrasonography, or echocardiography, we could not assess the exact association of genetic variation, hypoalbuminemia, vascular endothelial dysfunction, and hypertension. In addition, sex differences in albumin levels may have been attributed to hormonal basis for the differences between males and females or other factors such as oral contraceptives and menopause, but it is believed that the adjustment for sex would have reduced the error resulting from them in this study^28^. The Third, a social desirability bias could not be ruled out because medical history, use of medication, and consumption of tobacco or alcohol were all self-reported by subjects. This could have contributed to conflicting results with other studies. Finally, this GWAS could not reached a stringent genome-wide significance threshold (e.g. 10^–8^) and we could not perform a replication study owing to limited sample sizes of the cohort data and the lack of another independent cohort with similar phenotype. Moreover, the limited sample size of the hypoalbuminemia group could lead to bias in the nonlinear relationship between serum albumin and hypertension. This lack of sample size in this study is one of the major limitations and it raises the need for large-scale follow-up research. Because GWAS generally diminishes the ability to detect variants of small effects, a replication study is required to avoid false positives and to correctly assess the effect size of the SNP. As a result, large genotyping studies and robust replication studies are required in the future to confirm the associations and to find detailed relationships among the genotypic variants.

In conclusion, this study demonstrated that there was a causal relationship between hypoalbuminemia and the risk of future hypertension in the general population. The current findings may provide an opportunity to determine the underlying mechanisms of the effects of hypoalbuminemia on the increased risk of hypertension. Further replication studies and experimental studies are needed to clarify the causal mechanisms between hypoalbuminemia and incipient hypertension.

## Methods

### Study design and population

The present study used Ansan-Anseong cohort data from KoGES, in which biannual repeated surveys were collected between 2001 and 2014. The comprehensive profile and methods regarding the development of the KoGES have been presented previously^29^. Ansan-Anseong cohort data were developed to find the potential effect of candidate genetic variation on various chronic illnesses, which are from a medium-sized city (Ansan) and a rural area (Anseong) near Seoul, Korea. All participants underwent serial health examinations biannually including laboratory tests, electrocardiograms, chest X-rays, and health questionnaires. The use of antihypertensive therapy was assessed using an interviewer-administered questionnaire at baseline and every visit. All participants were enrolled voluntarily and provided written informed consent. All participants’ records, apart from the survey date and home region, were anonymized before analysis. This study was carried out in accordance with the Declaration of Helsinki and was approved by the Institutional Review Board (IRB) of Konkuk University Medical Center (IRB protocol: KUCH 2019-02-007). After the exclusion of subjects with missing data and those with hypertension, diabetes mellitus, chronic kidney disease, cardiovascular disease, and malignancy in KoGES, a total of 4325 eligible participants were categorized into two groups based on their serum albumin results (Supplementary Fig. S4).

### Anthropometric and clinical measurements

Anthropometric measurements were performed by well-trained examiners. Participants wore a lightweight gown or underwear. Height (Ht) was measured to the nearest 0.1 cm using a portable stadiometer (SECA 225, SECA, Hamburg Germany). Weight was measured to the nearest 0.1 kg on a calibrated balance-beam scale (GL-60000-20, CAS Korea, Seoul, Korea). Waist circumference was measured using a flexible tape at the narrowest point between the lowest border of the rib cage and the uppermost lateral border of the iliac crest at the end of normal expiration.

Trained examiners measured blood pressure (BP) according to a standardized protocol using an appropriate-sized cuff and a mercury sphygmomanometer at baseline and biannual follow-up visits. The first and fifth phases of Korotkoff sounds were used for SBP and DBP. BP measurements were repeated after 30-s intervals and were recorded to the nearest 2 mmHg. SBP and DBP were determined as the average of the right and left arm readings obtained three times between 7:00 a.m. and 9:00 a.m. after a minimum of 5 min of rest in a seated position; there were 5 min of rest between each measurement. Average rate of blood pressure change was calculated as the difference between blood pressure at baseline and last measurement just before the diagnosis of hypertension divided by the length of the period.

### Laboratory tests

Venous blood samples were collected after 8 h of overnight fasting. Fasting plasma concentrations of glucose, triglyceride (TG), high-density lipoprotein (HDL)-cholesterol, and low-density lipoprotein (LDL)-cholesterol were determined using a Hitachi Automatic Analyzer 7600 (Hitachi, Tokyo, Japan). Glycated hemoglobin (HbA1c) levels were determined by high-performance liquid chromatography (Variant II; BioRad Laboratories, Hercules, CA, USA). Serum creatinine levels were measured colorimetrically (Hitachi Automatic Analyzer 7600), and estimated glomerular filtration rate (eGFR) was calculated using the Chronic Kidney Disease Epidemiology Collaboration equation^30^. To obtain the urine albumin/creatinine ratio (UACR), urinary albumin was measured in spot urine using the immunoturbidimetric method, and urinary creatinine was measured using the colorimetric method.

### Genotyping

We analyzed the data on single nucleotide polymorphisms (SNPs) in the whole genome available to the research community through the Korean Association Resource (KARE) project from KoGES and used the Affymetrix Genome-Wide Human SNP Array 5.0 (Affymetrix Inc., Santa Clara, CA, USA) to genotype the samples from the Ansan-Anseong cohorts. The Bayesian Robust Linear Model with the Mahalanobis distance algorithm was used to determine the genotypes at SNP of Affymetrix 5.0. SNPs were excluded if any of the following criteria were met: (1) a call rate lower than 95%, (2) a minor allele frequency below 0.05, or (3) a significant deviation from the Hardy–Weinberg equilibrium below 0.001. Among the SNPs filtered by these criteria, only tagging SNPs were used for analysis in this study.

### Definition

According to previous epidemiologic studies^31–33^, we defined hypoalbuminemia as a serum albumin concentration of ≤ 4.0 g/dL. According to the Joint National Committee 7/8 guidelines^34,35^, hypertension was defined as either the use of antihypertensive therapy, SBP above 140 mmHg, or DBP above 90 mmHg.

### Statistical analysis

All data, including socio-demographic information, medical conditions, anthropometric and clinical measurements, and laboratory results, were presented as mean ± standard deviation or frequencies (and proportions). The normality of the distribution of parameters was analyzed using the Kolmogorov–Smirnov test. If the original data did not follow a Gaussian distribution, logarithmic transformation was applied to make the distribution more normal. Quantitative variables were compared using the Mann–Whitney U test, and categorical variables by the chi-square and Fisher's exact tests. The relationship between serum albumin and potential risk factors for chronic CVD was assessed by linear regression analysis. Hazard ratios (HRs) with 95% confidence intervals (CIs) were calculated by multiple Cox-proportional hazards models according to the development of hypertension (case vs. control). In order to compare hypertension-free survival between the groups, Kaplan–Meier analysis with the log-rank test was used. We used Supremum tests to evaluate proportional hazards assumptions.

Restricted cubic spline regression analysis was used to determine the possible non-linear dependency of the relation between candidate risk factor(s) and the risk of the dependent variable^36^.

Quantile–quantile plots and Manhattan plots were performed to visualize the results of the genome-wide association study, confirming the existence of relevant SNPs. We generated a regional association analysis using a web-based tool for the identification and annotation of proxy SNPs using HapMap (locuszoom.shp.umich.edu/locuszoom).

Because of the potential limitations of conventional statistical methods, an instrumental variable analysis (also known as Mendelian randomization) was applied to obtain causal inferences on the effect of an exposure on a clinically relevant outcome from observational data controlling for threats to its internal validity, including confounding variables, measurement error, spuriousness, simultaneity, and reverse causality^37–40^. In the two-stage least squares regression, the effect estimates from the second stage of the instrumental variable analysis and ordinary least square analysis were compared using the Durbin-Wu-Hausman test. Because conventional two-stage least squares regression analysis may have insufficient statistical power to assess the pathogenic relevance when there is a non-linear relationship between the exposure and outcome, further two-stage residual inclusion analysis was performed to draw inferences on the causal effects of candidate genetic variant(s) on the association between hypoalbuminemia and interesting phenotype (hypertension) while controlling for potential confounding factors^41–44^.

All statistical analyses were performed using PLINK version 1.09 (http://pngu.mgh.harvard.edu/~purcell/plink), R Statistical package software 3.2.2 (http://www.r-project.org), or Statistical Analysis Software (version 9.4; SAS Institute Inc., Cary, NC, USA).

## Supplementary Information

Supplementary Information 1.

Supplementary Information 2.
