# Supplementary material for: Genetically determined hypoalbuminemia as a risk factor for hypertension: instrumental variable analysis
Source: Sci Rep. 2021 May 28;11:11290. doi: 10.1038/s41598-021-89775-3 (PMC8163734; doi:10.1038/s41598-021-89775-3)
Supplement: Supplementary file 1 — Supplementary Information 1. [file 41598_2021_89775_MOESM1_ESM.pptx]

## Slide 1
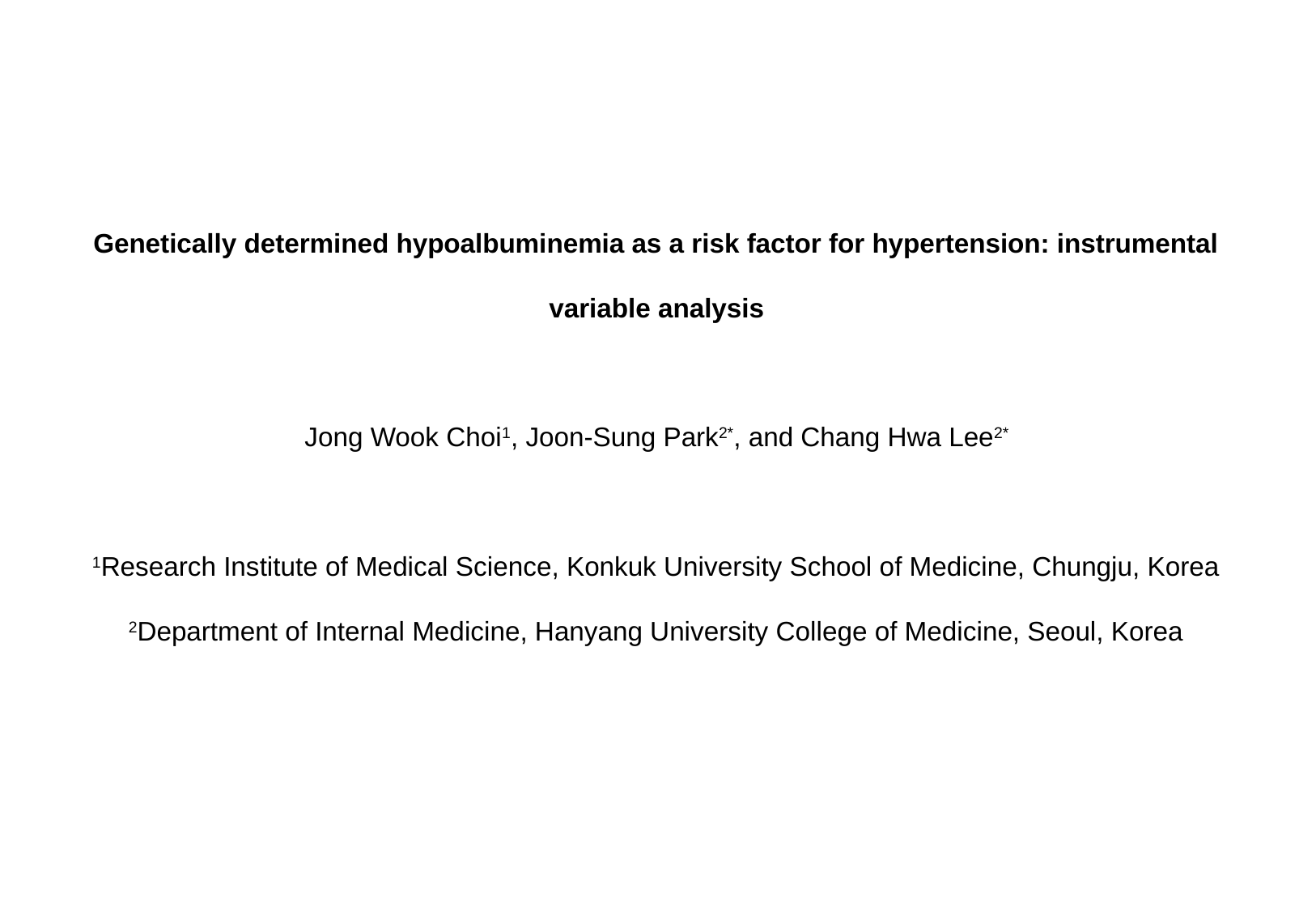

Genetically determined hypoalbuminemia as a risk factor for hypertension: instrumental variable analysis
Jong Wook Choi1, Joon-Sung Park2*, and Chang Hwa Lee2*
1Research Institute of Medical Science, Konkuk University School of Medicine, Chungju, Korea
2Department of Internal Medicine, Hanyang University College of Medicine, Seoul, Korea

## Slide 2
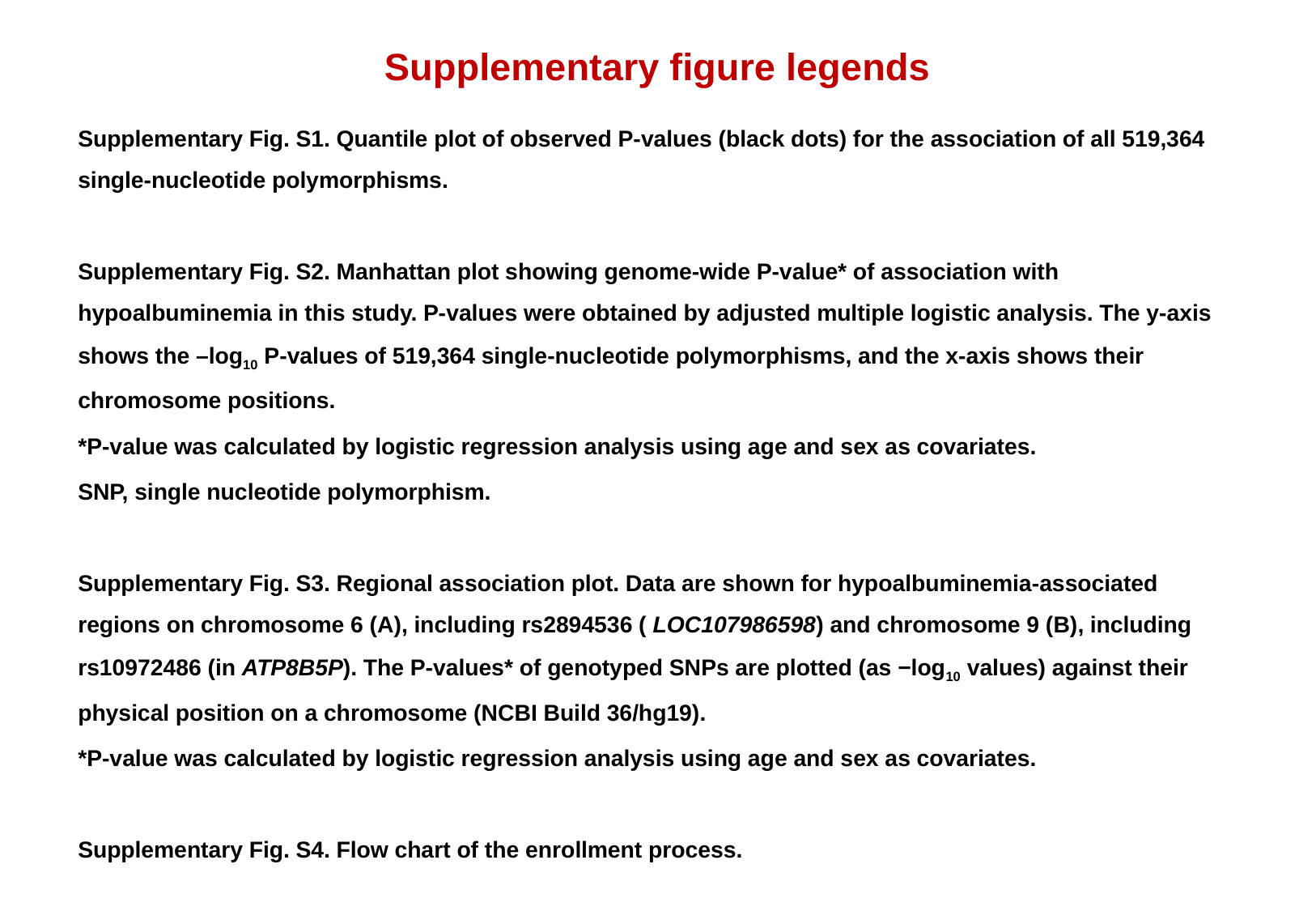

# Supplementary figure legends
Supplementary Fig. S1. Quantile plot of observed P-values (black dots) for the association of all 519,364 single-nucleotide polymorphisms.
Supplementary Fig. S2. Manhattan plot showing genome-wide P-value* of association with hypoalbuminemia in this study. P-values were obtained by adjusted multiple logistic analysis. The y-axis shows the –log10 P-values of 519,364 single-nucleotide polymorphisms, and the x-axis shows their chromosome positions.
*P-value was calculated by logistic regression analysis using age and sex as covariates.
SNP, single nucleotide polymorphism.
Supplementary Fig. S3. Regional association plot. Data are shown for hypoalbuminemia-associated regions on chromosome 6 (A), including rs2894536 ( LOC107986598) and chromosome 9 (B), including rs10972486 (in ATP8B5P). The P-values* of genotyped SNPs are plotted (as −log10 values) against their physical position on a chromosome (NCBI Build 36/hg19).
*P-value was calculated by logistic regression analysis using age and sex as covariates.
Supplementary Fig. S4. Flow chart of the enrollment process.

## Slide 3
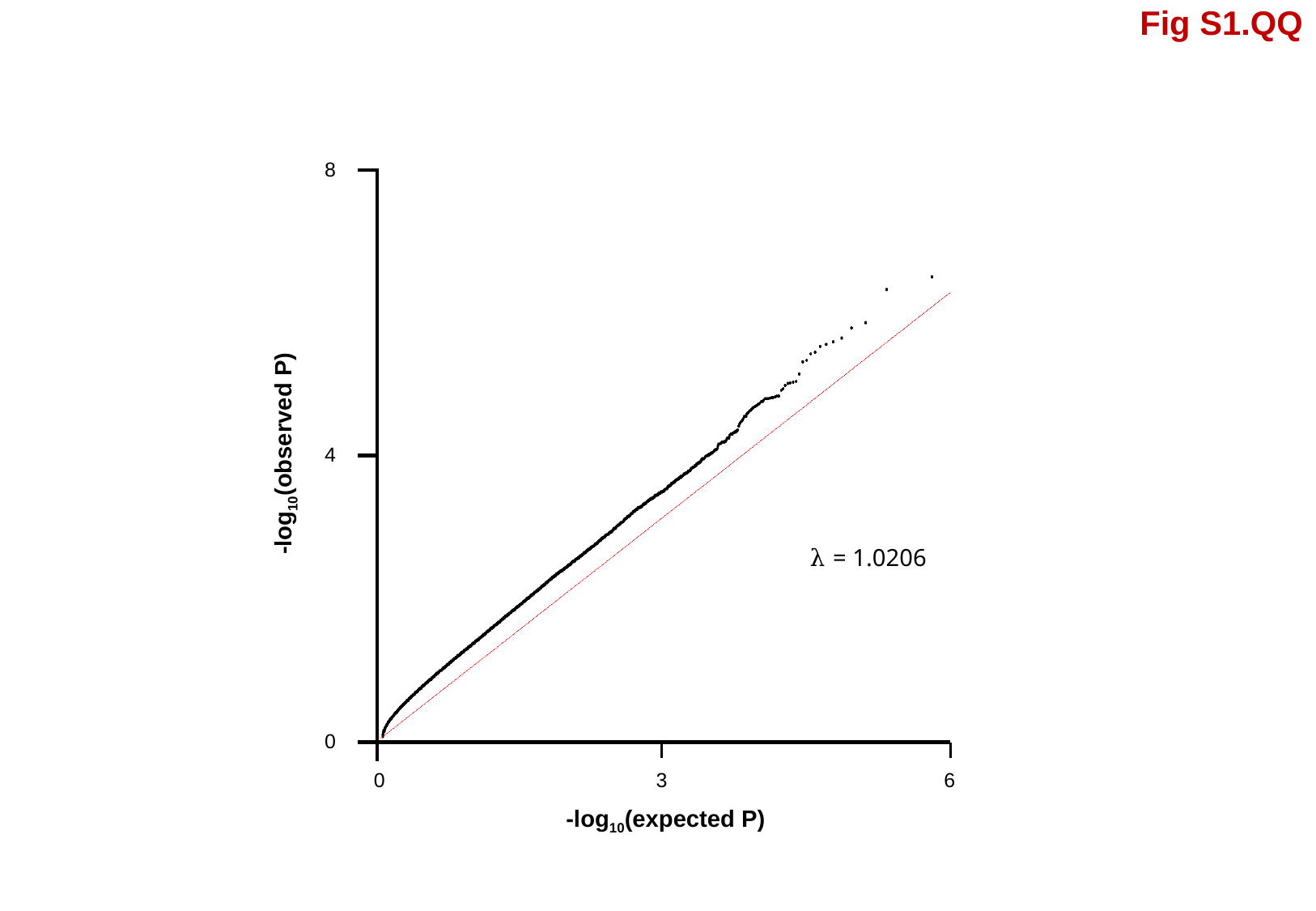

# Fig S1.QQ
8
4
0
-log10(observed P)
λ = 1.0206
0 3 6
-log10(expected P)

## Slide 4
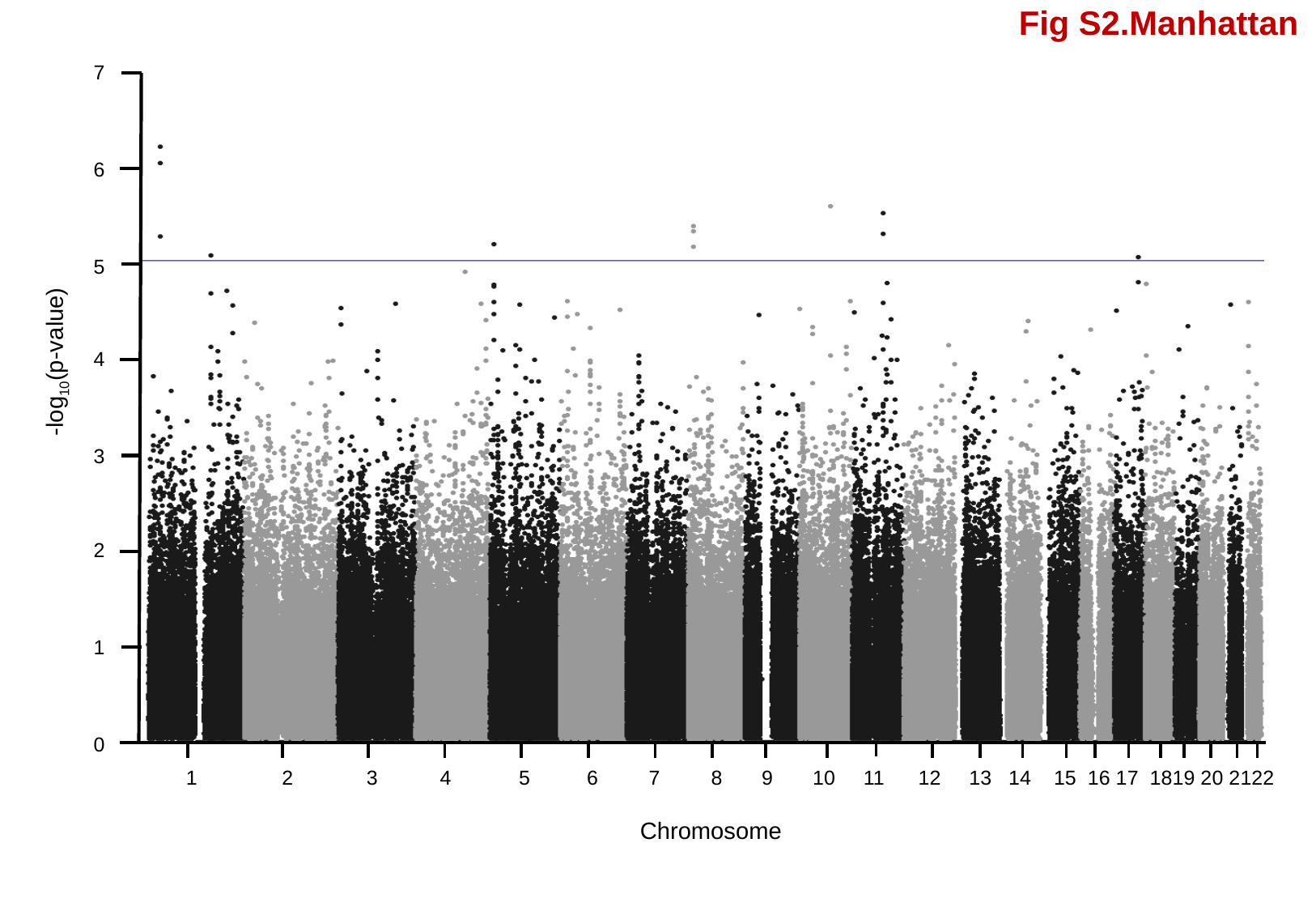

# Fig S2.Manhattan
7
6
5
4
3
2
1
0
-log10(p-value)
1 2 3 4 5 6 7 8 9 10 11 12 13 14 15 16 17 1819 20 2122
Chromosome

## Slide 5
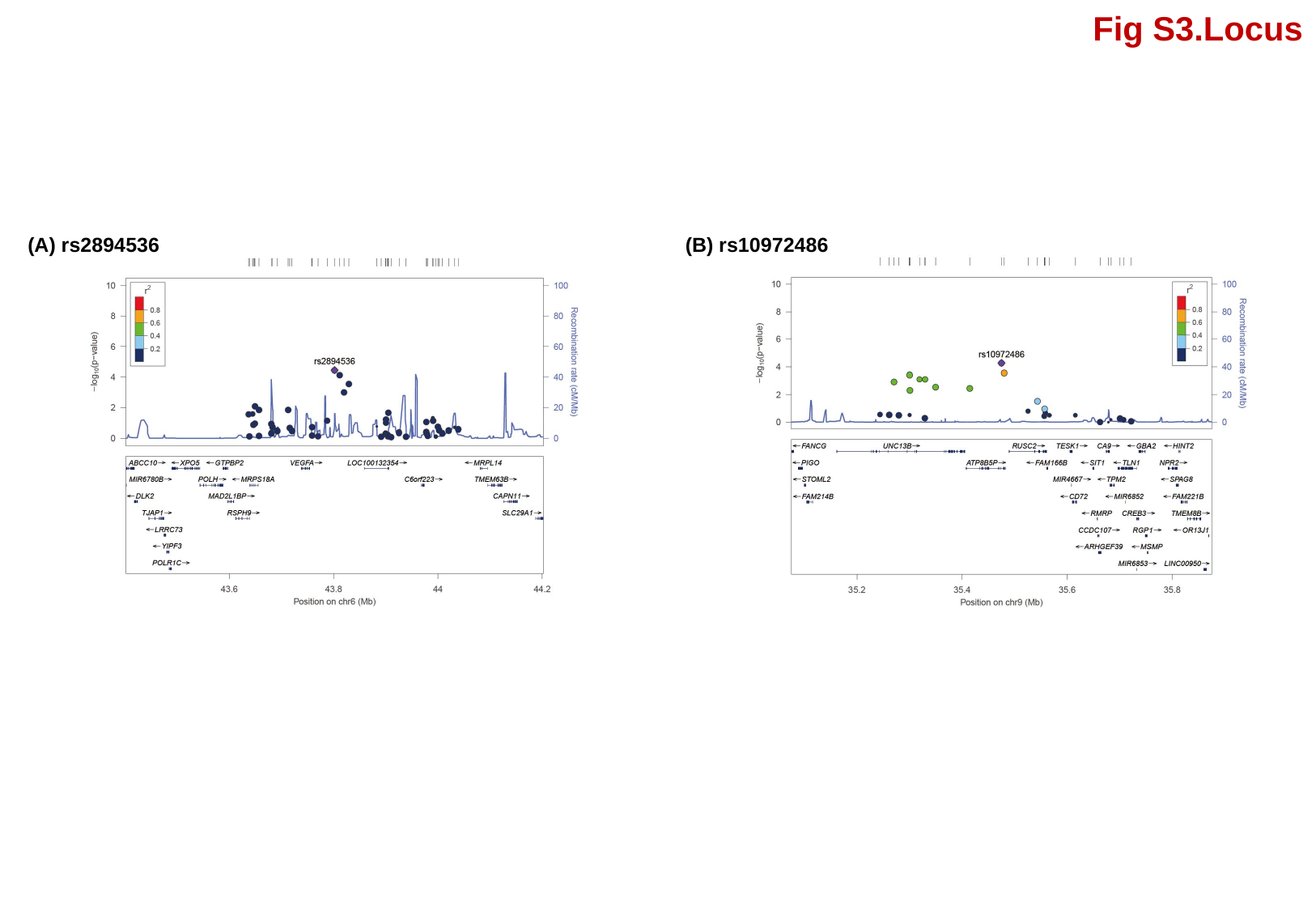

Fig S3.Locus
(A) rs2894536
(B) rs10972486

## Slide 6
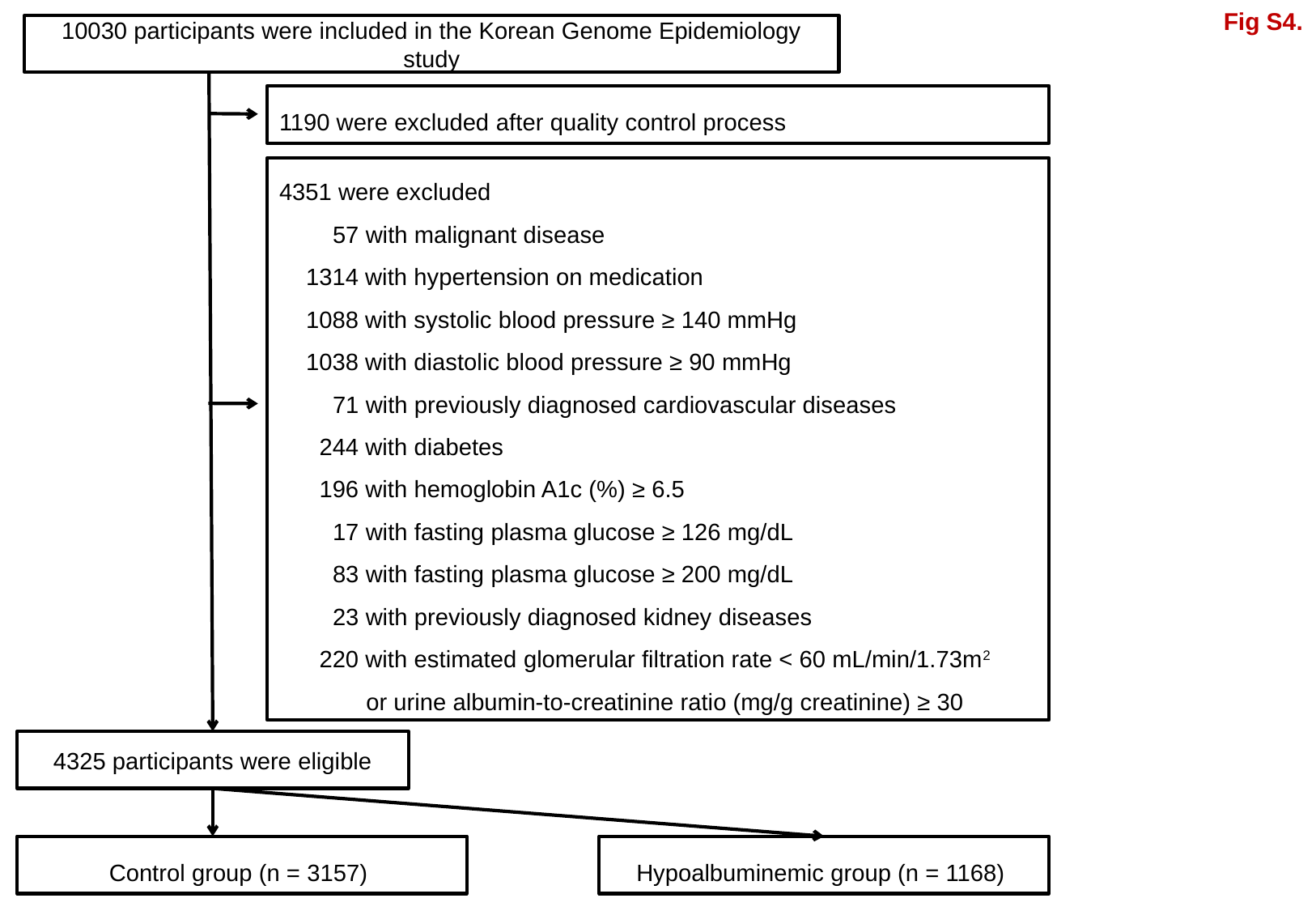

Fig S4.
10030 participants were included in the Korean Genome Epidemiology study
1190 were excluded after quality control process
4351 were excluded
 57 with malignant disease
 1314 with hypertension on medication
 1088 with systolic blood pressure ≥ 140 mmHg
 1038 with diastolic blood pressure ≥ 90 mmHg
 71 with previously diagnosed cardiovascular diseases
 244 with diabetes
 196 with hemoglobin A1c (%) ≥ 6.5
 17 with fasting plasma glucose ≥ 126 mg/dL
 83 with fasting plasma glucose ≥ 200 mg/dL
 23 with previously diagnosed kidney diseases
 220 with estimated glomerular filtration rate < 60 mL/min/1.73m2
 or urine albumin-to-creatinine ratio (mg/g creatinine) ≥ 30
4325 participants were eligible
Control group (n = 3157)
Hypoalbuminemic group (n = 1168)
