# Supplementary material for: Genetically determined hypoalbuminemia as a risk factor for hypertension: instrumental variable analysis
Source: Sci Rep. 2021 May 28;11:11290. doi: 10.1038/s41598-021-89775-3 (PMC8163734; doi:10.1038/s41598-021-89775-3)
Supplement: Supplementary file 2 — Supplementary Information 2. [file 41598_2021_89775_MOESM2_ESM.docx]

**Genetically determined hypoalbuminemia as a risk factor for hypertension: instrumental variable analysis**

Jong Wook Choi^1^, Joon-Sung Park^2*^, and Chang Hwa Lee^2*^

^1^Research Institute of Medical Science, Konkuk University School of Medicine, Chungju, Korea

^2^Department of Internal Medicine, Hanyang University College of Medicine, Seoul, Korea

Supplementary Table S1. Analysis of the association of the 71 single nucleotide polymorphisms (SNPs) with hypoalbuminemia^*^

| dbSNP ID | Nearest gene | Allele | Study  state | Genotype frequency (%) | | |  | | Additive | | | |  | | | Dominant | | | | |  | | | Recessive | | | | |  |  |
| --- | --- | --- | --- | --- | --- | --- | --- | --- | --- | --- | --- | --- | --- | --- | --- | --- | --- | --- | --- | --- | --- | --- | --- | --- | --- | --- | --- | --- | --- | --- |
|  |  | 1/2^**^ |  | 1 | 2 | | 3 |  | | OR^#^ | | P^#^ | | |  | | | OR^#^ | | P^#^ | |  | | | OR^#^ | | P^#^ | | | |
| rs3753613 | *HCRTR1* | C/T | Case | 47.3 | 44.2 | 8.5 |  | | 0.84 | | 8.0 x 10^-3^ | | |  | | | 0.72 | | 5.6 X 10^-6^ | | | |  | | | 0.80 | | 9.3 x 10^-2^ | |  |
|  |  |  | Control | 55.0 | 37.6 | 7.4 |  | |  | |  | | |  | | |  | |  | | | |  | | |  | |  | |  |
| rs2271933 | *HCRTR1* | C/T | Case | 49.6 | 41.7 | 8.7 |  | | 0.84 | | 1.1 X 10^-2^ | | |  | | | 0.70 | | 9.6 X 10^-7^ | | | |  | | | 0.82 | | 1.4 X 10^-1^ | |  |
|  |  |  | Control | 58.0 | 34.3 | 7.7 |  | |  | |  | | |  | | |  | |  | | | |  | | |  | |  | |  |
| rs4949454 | *PEF1* | A/G | Case | 48.3 | 43.5 | 8.2 |  | | 0.84 | | 1.3 X 10^-2^ | | |  | | | 0.70 | | 6.5 X 10^-7^ | | | |  | | | 0.83 | | 1.6 X 10^-1^ | |  |
|  |  |  | Control | 56.7 | 36.0 | 7.3 |  | |  | |  | | |  | | |  | |  | | | |  | | |  | |  | |  |
| rs3806368 | *RGS5* | A/G | Case | 50.1 | 41.2 | 8.7 |  | | 0.83 | | 6.5 X 10^-3^ | | |  | | | 0.73 | | 8.8 X 10^-6^ | | | |  | | | 0.78 | | 6.4 X 10^-2^ | |  |
|  |  |  | Control | 57.5 | 35.6 | 6.9 |  | |  | |  | | |  | | |  | |  | | | |  | | |  | |  | |  |
| rs1054943 | *RGS5* | G/C | Case | 50.0 | 41.3 | 8.7 |  | | 0.83 | | 6.9 X 10^-3^ | | |  | | | 0.74 | | 2.2 X 10^-5^ | | | |  | | | 0.78 | | 6.1 X 10^-2^ | |  |
|  |  |  | Control | 57.1 | 35.9 | 7.0 |  | |  | |  | | |  | | |  | |  | | | |  | | |  | |  | |  |
| rs9628673 | *LOC101928404,* | C/A | Case | 37.1 | 48.5 | 14.4 |  | | 0.83 | | 1.0 X 10^-3^ | | |  | | | 0.75 | | 8.0 X 10^-5^ | | | |  | | | 0.79 | | 2.6 X 10^-2^ | |  |
|  | *RGS5* |  | Control | 43.8 | 44.4 | 11.8 |  | |  | |  | | |  | | |  | |  | | | |  | | |  | |  | |  |
| rs2802809 | *NFASC* | G/A | Case | 26.4 | 48.8 | 24.8 |  | | 0.83 | | 2.1 X 10^-4^ | | |  | | | 0.88 | | 1.1 X 10^-1^ | | | |  | | | 0.69 | | 2.1 X 10^-5^ | |  |
|  |  |  | Control | 28.5 | 52.5 | 19.0 |  | |  | |  | | |  | | |  | |  | | | |  | | |  | |  | |  |
| rs13374930 | *LYPLAL1* | A/G | Case | 70.1 | 27.0 | 2.9 |  | | 1.02 | | 8.8 X 10^-1^ | | |  | | | 1.35 | | 5.7 X 10^-5^ | | | |  | | | 0.93 | | 7.4 X 10^-1^ | |  |
|  |  |  | Control | 63.1 | 34.1 | 2.8 |  | |  | |  | | |  | | |  | |  | | | |  | | |  | |  | |  |
| rs10863456 | *LYPLAL1* | A/G | Case | 69.8 | 27.3 | 2.9 |  | | 1.02 | | 8.5 X 10^-1^ | | |  | | | 1.36 | | 3.0 X 10^-5^ | | | |  | | | 0.94 | | 7.6 X 10^-1^ | |  |
|  |  |  | Control | 62.7 | 34.5 | 2.8 |  | |  | |  | | |  | | |  | |  | | | |  | | |  | |  | |  |
| rs780094 | *GCKR* | C/T | Case | 30.1 | 50.4 | 19.5 |  | | 1.19 | | 5.5 X 10^-4^ | | |  | | | 1.11 | | 1.7 X 10^-1^ | | | |  | | | 1.41 | | 4.4 X 10^-5^ | |  |
|  |  |  | Control | 27.7 | 46.8 | 25.5 |  | |  | |  | | |  | | |  | |  | | | |  | | |  | |  | |  |
| rs9860560 | *GRM7* | A/C | Case | 40.9 | 45.2 | 13.9 |  | | 0.79 | | 7.4 X 10^-5^ | | |  | | | 0.89 | | 1.0 X 10^-1^ | | | |  | | | 0.63 | | 4.6 X 10^-5^ | |  |
|  |  |  | Control | 43.7 | 46.7 | 9.6 |  | |  | |  | | |  | | |  | |  | | | |  | | |  | |  | |  |
| rs1504047 | *GRM7* | T/G | Case | 40.9 | 45.1 | 14.0 |  | | 0.78 | | 5.0 X 10^-5^ | | |  | | | 0.89 | | 9.3 X 10^-2^ | | | |  | | | 0.62 | | 3.1 X 10^-5^ | |  |
|  |  |  | Control | 43.7 | 46.7 | 9.6 |  | |  | |  | | |  | | |  | |  | | | |  | | |  | |  | |  |
| rs7628739 | intergenic | G/A | Case | 72.9 | 24.2 | 2.9 |  | | 0.51 | | 8.7 X 10^-5^ | | |  | | | 0.86 | | 5.6 X 10^-2^ | | | |  | | | 0.27 | | 1.0 X 10^-4^ | |  |
|  |  |  | Control | 75.7 | 23.4 | 0.9 |  | |  | |  | | |  | | |  | |  | | | |  | | |  | |  | |  |
| rs7624556 | intergenic | T/C | Case | 40.8 | 44.2 | 15.0 |  | | 1.16 | | 7.2 X 10^-3^ | | |  | | | 1.37 | | 2.8 X 10^-5^ | | | |  | | | 1.12 | | 2.4 X 10^-1^ | |  |
|  |  |  | Control | 34.7 | 49.7 | 15.6 |  | |  | |  | | |  | | |  | |  | | | |  | | |  | |  | |  |
| rs7692865 | intergenic | T/C | Case | 36.8 | 48.9 | 14.3 |  | | 0.92 | | 1.1 X 10^-1^ | | |  | | | 0.73 | | 1.3 X 10^-5^ | | | |  | | | 1.02 | | 8.7 X 10^-1^ | |  |
|  |  |  | Control | 43.4 | 42.0 | 14.6 |  | |  | |  | | |  | | |  | |  | | | |  | | |  | |  | |  |
| rs2131247 | intergenic | T/C | Case | 57.7 | 36.2 | 6.1 |  | | 1.25 | | 1.4 X 10^-3^ | | |  | | | 1.35 | | 2.8 X 10^-5^ | | | |  | | | 1.39 | | 2.4 X 10^-2^ | |  |
|  |  |  | Control | 51.3 | 40.5 | 8.2 |  | |  | |  | | |  | | |  | |  | | | |  | | |  | |  | |  |
| rs13105926 | intergenic | C/T | Case | 29.1 | 50.2 | 20.7 |  | | 1.18 | | 9.9 X 10^-4^ | | |  | | | 1.11 | | 1.9 X 10^-1^ | | | |  | | | 1.38 | | 8.2 X 10^-5^ | |  |
|  |  |  | Control | 26.7 | 46.9 | 26.4 |  | |  | |  | | |  | | |  | |  | | | |  | | |  | |  | |  |
| rs12505720 | intergenic | A/C | Case | 25.6 | 49.8 | 24.6 |  | | 0.82 | | 4.2 X 10^-5^ | | |  | | | 0.74 | | 7.7 X 10^-5^ | | | |  | | | 0.78 | | 4.1 X 10^-3^ | |  |
|  |  |  | Control | 31.6 | 47.6 | 20.8 |  | |  | |  | | |  | | |  | |  | | | |  | | |  | |  | |  |
| rs9313253 | intergenic | T/C | Case | 72.5 | 25.9 | 1.6 |  | | 1.63 | | 6.7 X 10^-6^ | | |  | | | 1.13 | | 1.2 X 10^-1^ | | | |  | | | 2.63 | | 7.6 X 10^-6^ | |  |
|  |  |  | Control | 69.7 | 26.5 | 3.8 |  | |  | |  | | |  | | |  | |  | | | |  | | |  | |  | |  |
| rs156477 | intergenic | T/C | Case | 71.4 | 26.7 | 1.9 |  | | 1.55 | | 2.7 X 10^-5^ | | |  | | | 1.13 | | 1.2 X 10^-1^ | | | |  | | | 2.38 | | 3.2 X 10^-5^ | |  |
|  |  |  | Control | 68.8 | 27.2 | 4.0 |  | |  | |  | | |  | | |  | |  | | | |  | | |  | |  | |  |
| rs13356951 | *LOC101929307* | C/T | Case | 72.2 | 26.0 | 1.8 |  | | 1.54 | | 3.6 X 10^-5^ | | |  | | | 1.12 | | 1.5 X 10^-1^ | | | |  | | | 2.35 | | 4.0 X 10^-5^ | |  |
|  |  |  | Control | 69.6 | 26.5 | 3.9 |  | |  | |  | | |  | | |  | |  | | | |  | | |  | |  | |  |
| rs999428 | *LOC101929307* | C/T | Case | 72.1 | 26.1 | 1.8 |  | | 1.57 | | 1.8 X 10^-5^ | | |  | | | 1.11 | | 1.7 X 10^-1^ | | | |  | | | 2.46 | | 1.9 X 10^-5^ | |  |
|  |  |  | Control | 69.6 | 26.5 | 3.9 |  | |  | |  | | |  | | |  | |  | | | |  | | |  | |  | |  |
| rs999427 | *LOC101929307* | A/G | Case | 72.4 | 25.7 | 1.9 |  | | 1.52 | | 6.6 X 10^-5^ | | |  | | | 1.11 | | 2.0 X 10^-1^ | | | |  | | | 2.30 | | 6.8 X 10^-5^ | |  |
|  |  |  | Control | 70.1 | 26.0 | 3.9 |  | |  | |  | | |  | | |  | |  | | | |  | | |  | |  | |  |
| rs2963394 | *LOC101929307* | T/A | Case | 71.6 | 26.6 | 1.8 |  | | 1.58 | | 1.9 X 10^-5^ | | |  | | | 1.13 | | 1.1 X 10^-1^ | | | |  | | | 2.45 | | 2.2 X 10^-5^ | |  |
|  |  |  | Control | 68.7 | 27.4 | 3.9 |  | |  | |  | | |  | | |  | |  | | | |  | | |  | |  | |  |
| rs16891241 | intergenic | G/A | Case | 26.1 | 51.0 | 22.9 |  | | 0.86 | | 3.5 X 10^-3^ | | |  | | | 0.74 | | 8.5 X 10^-5^ | | | |  | | | 0.91 | | 2.6 X 10^-1^ | |  |
|  |  |  | Control | 31.9 | 46.9 | 21.2 |  | |  | |  | | |  | | |  | |  | | | |  | | |  | |  | |  |
| rs11953193 | *MAST4* | C/A | Case | 78.9 | 19.4 | 1.7 |  | | 1.10 | | 5.1 X 10^-1^ | | |  | | | 1.39 | | 7.6 X 10^-5^ | | | |  | | | 1.11 | | 7.0 X 10^-1^ | |  |
|  |  |  | Control | 73.1 | 25.2 | 1.7 |  | |  | |  | | |  | | |  | |  | | | |  | | |  | |  | |  |
| rs6870971 | *SV2C* | T/C | Case | 37.3 | 48.8 | 13.9 |  | | 0.93 | | 2.1 X 10^-1^ | | |  | | | 0.74 | | 2.9 X 10^-5^ | | | |  | | | 1.05 | | 6.2 X 10^-1^ | |  |
|  |  |  | Control | 43.3 | 42.0 | 14.4 |  | |  | |  | | |  | | |  | |  | | | |  | | |  | |  | |  |
| rs10036293 | *SV2C* | G/C | Case | 40.0 | 47.2 | 12.8 |  | | 0.95 | | 3.5 X 10^-1^ | | |  | | | 0.75 | | 8.3 X 10^-5^ | | | |  | | | 1.07 | | 5.5 X 10^-1^ | |  |
|  |  |  | Control | 46.2 | 40.5 | 13.3 |  | |  | |  | | |  | | |  | |  | | | |  | | |  | |  | |  |
| rs2923173 | intergenic | A/T | Case | 61.2 | 34.1 | 4.7 |  | | 1.35 | | 3.9 X 10^-5^ | | |  | | | 1.28 | | 6.4 X 10^-4^ | | | |  | | | 1.71 | | 2.0 X 10^-4^ | |  |
|  |  |  | Control | 56.1 | 36.3 | 7.6 |  | |  | |  | | |  | | |  | |  | | | |  | | |  | |  | |  |
| rs6910353 | intergenic | A/C | Case | 36.2 | 49.1 | 14.7 |  | | 1.35 | | 3.9 X 10^-5^ | | |  | | | 1.28 | | 6.4 X 10^-4^ | | | |  | | | 1.71 | | 2.0 X 10^-4^ | |  |
|  |  |  | Control | 32.5 | 48.7 | 18.8 |  | |  | |  | | |  | | |  | |  | | | |  | | |  | |  | |  |
| rs12194394 | intergenic | A/G | Case | 36.8 | 48.5 | 14.7 |  | | 1.24 | | 3.8 X 10^-5^ | | |  | | | 1.24 | | 5.0 X 10^-3^ | | | |  | | | 1.43 | | 1.7 X 10^-4^ | |  |
|  |  |  | Control | 33.0 | 48.2 | 18.8 |  | |  | |  | | |  | | |  | |  | | | |  | | |  | |  | |  |
| rs1536041 | *ITPR3* | A/G | Case | 76.7 | 21.7 | 1.6 |  | | 1.08 | | 5.6 X 10^-1^ | | |  | | | 1.37 | | 8.2 X 10^-5^ | | | |  | | | 1.08 | | 7.7 X 10^-1^ | |  |
|  |  |  | Control | 71.6 | 26.6 | 1.8 |  | |  | |  | | |  | | |  | |  | | | |  | | |  | |  | |  |
| rs2894536 | *LOC107986598* | G/A | Case | 76.1 | 22.5 | 1.4 |  | | 1.62 | | 3.6 X 10^-5^ | | |  | | | 1.27 | | 2.3 X 10^-3^ | | | |  | | | 2.52 | | 7.4 X 10^-5^ | |  |
|  |  |  | Control | 71.1 | 25.6 | 3.3 |  | |  | |  | | |  | | |  | |  | | | |  | | |  | |  | |  |
| rs10943468 | intergenic | G/A | Case | 80.5 | 18.6 | 0.9 |  | | 1.07 | | 7.0 X 10^-1^ | | |  | | | 1.41 | | 5.0 X 10^-5^ | | | |  | | | 1.07 | | 8.6 X 10^-1^ | |  |
|  |  |  | Control | 74.9 | 24.1 | 1.0 |  | |  | |  | | |  | | |  | |  | | | |  | | |  | |  | |  |
| rs687694 | intergenic | G/T | Case | 48.9 | 41.7 | 9.4 |  | | 1.15 | | 2.5 X 10^-2^ | | |  | | | 1.35 | | 3.3 X 10^-5^ | | | |  | | | 1.14 | | 2.8 X 10^-1^ | |  |
|  |  |  | Control | 42.2 | 47.3 | 10.5 |  | |  | |  | | |  | | |  | |  | | | |  | | |  | |  | |  |
| rs6977000 | *PDE1C* | A/G | Case | 69.6 | 28.1 | 2.3 |  | | 1.45 | | 9.8 X 10^-5^ | | |  | | | 1.17 | | 4.3 X 10^-2^ | | | |  | | | 2.06 | | 1.5 X 10^-4^ | |  |
|  |  |  | Control | 66.6 | 28.9 | 4.5 |  | |  | |  | | |  | | |  | |  | | | |  | | |  | |  | |  |
| rs4831521 | intergenic | G/A | Case | 43.1 | 46.4 | 10.5 |  | | 0.82 | | 1.6 X 10^-3^ | | |  | | | 0.73 | | 7.1 X 10^-6^ | | | |  | | | 0.77 | | 3.4 X 10^-2^ | |  |
|  |  |  | Control | 51.1 | 40.7 | 8.2 |  | |  | |  | | |  | | |  | |  | | | |  | | |  | |  | |  |
| rs2410138 | intergenic | G/A | Case | 43.1 | 46.5 | 10.4 |  | | 0.81 | | 1.1 X 10^-3^ | | |  | | | 0.72 | | 4.9 X 10^-6^ | | | |  | | | 0.75 | | 2.5 X 10^-2^ | |  |
|  |  |  | Control | 51.1 | 40.8 | 8.1 |  | |  | |  | | |  | | |  | |  | | | |  | | |  | |  | |  |
| rs2898379 | intergenic | A/T | Case | 43.1 | 46.5 | 10.4 |  | | 0.81 | | 1.3 X 10^-3^ | | |  | | | 0.72 | | 4.4 X 10^-6^ | | | |  | | | 0.76 | | 3.1 X 10^-2^ | |  |
|  |  |  | Control | 51.2 | 40.7 | 8.1 |  | |  | |  | | |  | | |  | |  | | | |  | | |  | |  | |  |
| rs10972486 | *ATP8B5P* | T/C | Case | 44.2 | 45.5 | 10.3 |  | | 1.25 | | 5.3 X 10^-5^ | | |  | | | 1.14 | | 7.4 X 10^-2^ | | | |  | | | 1.54 | | 3.7 X 10^-5^ | |  |
|  |  |  | Control | 41.4 | 43.7 | 14.9 |  | |  | |  | | |  | | |  | |  | | | |  | | |  | |  | |  |
| rs7898120 | *ADARB2* | T/G | Case | 60.5 | 35.1 | 4.4 |  | | 1.36 | | 3.2 X 10^-5^ | | |  | | | 1.17 | | 3.0 X 10^-2^ | | | |  | | | 1.81 | | 5.4 X 10^-5^ | |  |
|  |  |  | Control | 57.1 | 35.4 | 7.5 |  | |  | |  | | |  | | |  | |  | | | |  | | |  | |  | |  |
| rs722987 | intergenic | G/C | Case | 42.6 | 44.6 | 12.8 |  | | 1.13 | | 2.8 X 10^-2^ | | |  | | | 1.35 | | 4.9 X 10^-5^ | | | |  | | | 1.08 | | 4.6 X 10^-5^ | |  |
|  |  |  | Control | 35.9 | 50.2 | 13.9 |  | |  | |  | | |  | | |  | |  | | | |  | | |  | |  | |  |
| rs881423 | intergenic | A/G | Case | 42.5 | 44.9 | 12.6 |  | | 1.12 | | 5.2 X 10^-2^ | | |  | | | 1.34 | | 5.8 X 10^-5^ | | | |  | | | 1.05 | | 6.5 X 10^-1^ | |  |
|  |  |  | Control | 35.8 | 50.8 | 13.4 |  | |  | |  | | |  | | |  | |  | | | |  | | |  | |  | |  |
| rs16936709 | *ZMIZ1-AS1* | C/T | Case | 81.9 | 17.1 | 1.0 |  | | 0.69 | | 9.9 X 10^-2^ | | |  | | | 0.68 | | 9.8 X 10^-5^ | | | |  | | | 0.50 | | 1.3 X 10^-5^ | |  |
|  |  |  | Control | 86.6 | 12.9 | 0.5 |  | |  | |  | | |  | | |  | |  | | | |  | | |  | |  | |  |
| rs2420656 | intergenic | C/T | Case | 79.1 | 19.6 | 1.3 |  | | 1.09 | | 5.8 X 10^-1^ | | |  | | | 1.39 | | 8.0 X 10^-5^ | | | |  | | | 1.10 | | 7.6 X 10^-5^ | |  |
|  |  |  | Control | 74.2 | 24.4 | 1.4 |  | |  | |  | | |  | | |  | |  | | | |  | | |  | |  | |  |
| rs10788010 | intergenic | T/C | Case | 79.2 | 19.5 | 1.3 |  | | 1.10 | | 5.5 X 10^-1^ | | |  | | | 1.38 | | 9.3 X 10^-5^ | | | |  | | | 1.12 | | 7.2 X 10^-1^ | |  |
|  |  |  | Control | 74.4 | 24.2 | 1.4 |  | |  | |  | | |  | | |  | |  | | | |  | | |  | |  | |  |
| rs10734089 | *LOC107984280* | G/C | Case | 78.6 | 20.1 | 1.3 |  | | 1.37 | | 1.7 X 10^-2^ | | |  | | | 1.41 | | 2.6 X 10^-5^ | | | |  | | | 1.74 | | 3.3 X 10^-2^ | |  |
|  |  |  | Control | 73.3 | 24.4 | 2.3 |  | |  | |  | | |  | | |  | |  | | | |  | | |  | |  | |  |
| rs17257728 | intergenic | T/C | Case | 76.5 | 21.9 | 1.6 |  | | 1.04 | | 7.9 X 10^-1^ | | |  | | | 0.69 | | 3.5 X 10^-5^ | | | |  | | | 1.16 | | 5.8 X 10^-1^ | |  |
|  |  |  | Control | 81.8 | 16.5 | 1.7 |  | |  | |  | | |  | | |  | |  | | | |  | | |  | |  | |  |
| rs12271212 | intergenic | A/G | Case | 63.5 | 32.4 | 4.1 |  | | 0.84 | | 8.0 X 10^-2^ | | |  | | | 0.73 | | 6.0 X 10^-5^ | | | |  | | | 0.78 | | 2.0 X 10^-1^ | |  |
|  |  |  | Control | 70.4 | 26.3 | 3.3 |  | |  | |  | | |  | | |  | |  | | | |  | | |  | |  | |  |
| rs1955011 | *LOC107984423,* | T/C | Case | 50.2 | 40.9 | 8.9 |  | | 1.18 | | 1.0 X 10^-3^ | | |  | | | 1.39 | | 3.2 X 10^-6^ | | | |  | | | 1.18 | | 1.8 X 10^-1^ | |  |
|  | *MIR4300HG* |  | Control | 42.8 | 47.3 | 9.9 |  | |  | |  | | |  | | |  | |  | | | |  | | |  | |  | |  |
| rs645809 | *MIR4300HG* | A/G | Case | 30.3 | 50.1 | 19.6 |  | | 1.24 | | 2.8 X 10^-1^ | | |  | | | 1.30 | | 1.3 X 10^-3^ | | | |  | | | 1.36 | | 3.3 X 10^-4^ | |  |
|  |  |  | Control | 25.4 | 50.4 | 24.2 |  | |  | |  | | |  | | |  | |  | | | |  | | |  | |  | |  |
| rs896993 | *MIR4300HG* | C/G | Case | 81.0 | 18.1 | 0.9 |  | | 1.00 | | 9.9 X 10^-1^ | | |  | | | 1.48 | | 5.2 X 10^-6^ | | | |  | | | 0.92 | | 8.2 X 10^-1^ | |  |
|  |  |  | Control | 74.6 | 24.5 | 0.9 |  | |  | |  | | |  | | |  | |  | | | |  | | |  | |  | |  |
| rs2373947 | *MIR4300HG* | C/G | Case | 84.7 | 14.5 | 0.8 |  | | 0.68 | | 1.3 X 10^-1^ | | |  | | | 1.43 | | 8.4 X 10^-5^ | | | |  | | | 0.44 | | 9.6 X 10^-2^ | |  |
|  |  |  | Control | 79.7 | 19.9 | 0.4 |  | |  | |  | | |  | | |  | |  | | | |  | | |  | |  | |  |
| rs16924259 | *DISC1FP1* | A/G | Case | 64.3 | 32.3 | 3.4 |  | | 1.40 | | 6.2 X 10^-5^ | | |  | | | 1.17 | | 3.1 X 10^-2^ | | | |  | | | 1.90 | | 1.1 X 10^-4^ | |  |
|  |  |  | Control | 60.5 | 33.6 | 2.9 |  | |  | |  | | |  | | |  | |  | | | |  | | |  | |  | |  |
| rs10501745 | *DISC1FP1* | T/C | Case | 66.7 | 29.9 | 3.4 |  | | 1.42 | | 1.7 X 10^-5^ | | |  | | | 1.16 | | 4.1 X 10^-2^ | | | |  | | | 1.98 | | 2.5 X 10^-5^ | |  |
|  |  |  | Control | 63.2 | 30.6 | 6.2 |  | |  | |  | | |  | | |  | |  | | | |  | | |  | |  | |  |
| rs4754017 | Intergenic | T/A | Case | 40.7 | 47.4 | 11.9 |  | | 1.23 | | 1.6 X 10^-4^ | | |  | | | 1.10 | | 2.1 X 10^-1^ | | | |  | | | 1.51 | | 4.1 X 10^-5^ | |  |
|  |  |  | Control | 38.6 | 45.1 | 16.3 |  | |  | |  | | |  | | |  | |  | | | |  | | |  | |  | |  |
| rs1647103 | Intergenic | T/C | Case | 56.1 | 37.2 | 6.7 |  | | 1.30 | | 7.5 X 10^-5^ | | |  | | | 1.26 | | 1.2 X 10^-3^ | | | |  | | | 1.56 | | 4.3 X 10^-4^ | |  |
|  |  |  | Control | 50.4 | 39.5 | 10.1 |  | |  | |  | | |  | | |  | |  | | | |  | | |  | |  | |  |
| rs6573780 | *GPHN,* | A/C | Case | 29.5 | 48.3 | 22.2 |  | | 1.15 | | 6.1 X 10^-3^ | | |  | | | 1.39 | | 5.4 X 10^-5^ | | | |  | | | 1.05 | | 5.6 X 10^-1^ | |  |
|  | *PLEKHH1* |  | Control | 23.7 | 52.6 | 23.7 |  | |  | |  | | |  | | |  | |  | | | |  | | |  | |  | |  |
| rs42923 | *HEATR4* | C/T | Case | 80.6 | 18.3 | 1.1 |  | | 1.59 | | 3.4 X 10^-4^ | | |  | | | 1.41 | | 4.3 X 10^-5^ | | | |  | | | 2.39 | | 7.8 X 10^-4^ | |  |
|  |  |  | Control | 74.8 | 22.5 | 2.7 |  | |  | |  | | |  | | |  | |  | | | |  | | |  | |  | |  |
| rs10220852 | *LOC105370826,* | T/C | Case | 51.5 | 38.9 | 9.6 |  | | 1.17 | | 1.2 X 10^-2^ | | |  | | | 1.32 | | 1.0 X 10^-4^ | | | |  | | | 1.20 | | 1.2 X 10^-1^ | |  |
|  | *WDR72* |  | Control | 44.5 | 44.1 | 11.4 |  | |  | |  | | |  | | |  | |  | | | |  | | |  | |  | |  |
| rs17516321 | *GSG1L* | T/C | Case | 54.2 | 39.3 | 6.5 |  | | 1.30 | | 5.2 X 10^-5^ | | |  | | | 1.26 | | 1.0 X 10^-3^ | | | |  | | | 1.57 | | 3.4 X 10^-4^ | |  |
|  |  |  | Control | 48.8 | 41.3 | 9.9 |  | |  | |  | | |  | | |  | |  | | | |  | | |  | |  | |  |
| rs8070008 | *DHX33* | G/C | Case | 40.5 | 47.5 | 12.0 |  | | 0.88 | | 2.4 X 10^-2^ | | |  | | | 0.74 | | 3.3 X 10^-5^ | | | |  | | | 0.89 | | 3.1 X 10^-1^ | |  |
|  |  |  | Control | 47.7 | 41.5 | 10.8 |  | |  | |  | | |  | | |  | |  | | | |  | | |  | |  | |  |
| rs11871336 | Intergenic | C/T | Case | 28.3 | 50.5 | 21.2 |  | | 1.24 | | 1.8 X 10^-5^ | | |  | | | 1.23 | | 1.1 X 10^-2^ | | | |  | | | 1.44 | | 9.1 X 10^-6^ | |  |
|  |  |  | Control | 24.4 | 48.4 | 27.2 |  | |  | |  | | |  | | |  | |  | | | |  | | |  | |  | |  |
| rs2109448 | Intergenic | A/G | Case | 29.8 | 48.9 | 21.3 |  | | 0.80 | | 1.7 X 10^-5^ | | |  | | | 0.73 | | 2.5 X 10^-5^ | | | |  | | | 0.75 | | 1.9 X 10^-3^ | |  |
|  |  |  | Control | 36.0 | 46.7 | 17.3 |  | |  | |  | | |  | | |  | |  | | | |  | | |  | |  | |  |
| rs8090956 | *MYOM1* | A/G | Case | 27.2 | 49.8 | 23.0 |  | | 1.12 | | 2.8 X 10^-2^ | | |  | | | 1.39 | | 9.7 X 10^-5^ | | | |  | | | 0.98 | | 7.8 X 10^-1^ | |  |
|  |  |  | Control | 21.5 | 55.7 | 22.8 |  | |  | |  | | |  | | |  | |  | | | |  | | |  | |  | |  |
| rs4798160 | *DLGAP1* | A/G | Case | 38.4 | 48.8 | 12.8 |  | | 1.22 | | 3.0 X 10^-4^ | | |  | | | 1.05 | | 5.2 X 10^-1^ | | | |  | | | 1.53 | | 1.8 X 10^-5^ | |  |
|  |  |  | Control | 37.7 | 44.7 | 17.6 |  | |  | |  | | |  | | |  | |  | | | |  | | |  | |  | |  |
| rs8099884 | Intergenic | C/A | Case | 27.7 | 48.8 | 23.5 |  | | 0.82 | | 8.5 X 10^-5^ | | |  | | | 0.80 | | 3.7 X 10^-3^ | | | |  | | | 0.73 | | 4.8 X 10^-4^ | |  |
|  |  |  | Control | 32.3 | 49.1 | 18.6 |  | |  | |  | | |  | | |  | |  | | | |  | | |  | |  | |  |
| rs10413028 | Intergenic | C/T | Case | 30.7 | 51.2 | 18.1 |  | | 1.17 | | 2.0 X 10^-3^ | | |  | | | 1.04 | | 6.0 X 10^-1^ | | | |  | | | 1.42 | | 4.8 X 10^-5^ | |  |
|  |  |  | Control | 30.4 | 47.0 | 22.6 |  | |  | |  | | |  | | |  | |  | | | |  | | |  | |  | |  |
| rs2824571 | *CHODL* | T/C | Case | 27.6 | 50.3 | 22.1 |  | | 0.89 | | 1.6 X 10^-2^ | | |  | | | 0.73 | | 3.1 X 10^-5^ | | | |  | | | 0.98 | | 8.3 X 10^-1^ | |  |
|  |  |  | Control | 33.7 | 44.3 | 22.0 |  | |  | |  | | |  | | |  | |  | | | |  | | |  | |  | |  |
| rs361594 | *LOC101929426* | A/G | Case | 28.7 | 50.2 | 21.1 |  | | 1.22 | | 7.7 X 10^-5^ | | |  | | | 1.33 | | 3.1 X 10^-4^ | | | |  | | | 1.28 | | 2.8 X 10^-3^ | |  |
|  |  |  | Control | 23.6 | 51.1 | 25.3 |  | |  | |  | | |  | | |  | |  | | | |  | | |  | |  | |  |
| rs362250 | Intergenic | T/C | Case | 24.8 | 49.7 | 25.5 |  | | 0.81 | | 2.7 X 10^-5^ | | |  | | | 0.80 | | 4.5 X 10^-3^ | | | |  | | | 0.71 | | 7.0 X 10^-5^ | |  |
|  |  |  | Control | 29.0 | 50.9 | 20.1 |  | |  | |  | | |  | | |  | |  | | | |  | | |  | |  | |  |

OR, Odds ratio.

^*^Defined as serum albumin concentration of 4.0 g/dL or less.

^**^Allele 1, dominant allele; allele 2, non- dominant allele.

^#^Calculated by logistic regression analysis with age and sex as covariates.

Supplementary Table S2. Relationship of serum albumin (endogenous variable) with the change in (A) systolic blood pressure (ΔSBP) and diastolic blood pressure (ΔDBP) as tested by both ordinary least squares linear regression and the application of two-stage least squares regression analysis using candidate genetic polymorphisms as an instrument variable

|  | ΔSBP (mm Hg/month) | | | | |  | ΔDBP (mm Hg/month) | | | | | | | | | |
| --- | --- | --- | --- | --- | --- | --- | --- | --- | --- | --- | --- | --- | --- | --- | --- | --- |
| Instrumental variable | β | SE | P | F | P_DWH_^*^ |  | β | SE | | P | | F | | | P_DWH_^*^ |  |
| Ordinary least square linear regression | | | | | | | | | | | | | | | | |
| Albumin (g/dL) | -0.043 | 0.014 | 0.0017 |  |  |  | -0.103 | | 0.017 | | <0.0001 | |  |  | | |
| Two-stage least squares regression analysis | | | | | | | | | | | | | | | | |
| rs3753613 (vs. CC) | -0.050 | 0.007 | <0.0001 | 16.37 | 0.0010 |  | 0.001 | | 0.008 | | 0.9593 | | 5.14 | 0.1620 | | |
| rs2271933 (vs. CC) | -0.051 | 0.007 | <0.0001 | 15.67 | 0.0013 |  | 0.001 | | 0.009 | | 0.9716 | | 5.04 | 0.1686 | | |
| rs4949454 (vs. AA) | -0.051 | 0.007 | <0.0001 | 16.37 | 0.0010 |  | 0.001 | | 0.008 | | 0.9484 | | 5.13 | 0.1624 | | |
| rs3806368 (vs. AA) | -0.050 | 0.007 | <0.0001 | 16.41 | 0.0009 |  | 0.001 | | 0.008 | | 0.9593 | | 5.08 | 0.1657 | | |
| rs1054943 (vs. GG) | -0.050 | 0.007 | <0.0001 | 16.91 | 0.0007 |  | 0.001 | | 0.008 | | 0.9593 | | 4.43 | 0.2182 | | |
| rs9628673 (vs. CC) | -0.050 | 0.007 | <0.0001 | 16.50 | 0.0009 |  | 0.001 | | 0.008 | | 0.9593 | | 5.04 | 0.1689 | | |
| rs2802809 (vs. GG) | -0.049 | 0.007 | <0.0001 | 16.46 | 0.0009 |  | 0.002 | | 0.008 | | 0.8317 | | 5.01 | 0.1709 | | |
| rs13374930 (vs. AA) | -0.050 | 0.007 | <0.0001 | 16.57 | 0.0009 |  | -0.001 | | 0.008 | | 0.9600 | | 5.08 | 0.1660 | | |
| rs10863456 (vs. AA) | -0.050 | 0.007 | <0.0001 | 16.57 | 0.0009 |  | -0.001 | | 0.008 | | 0.9600 | | 5.08 | 0.1660 | | |
| rs780094 (vs. CC) | -0.052 | 0.007 | <0.0001 | 16.71 | 0.0008 |  | -0.001 | | 0.008 | | 0.9428 | | 4.96 | 0.1750 | | |
| rs9860560 (vs. AA) | -0.050 | 0.007 | <0.0001 | 17.45 | 0.0006 |  | 0.001 | | 0.008 | | 0.9600 | | 4.66 | 0.1987 | | |
| rs1504047 (vs. TT) | -0.050 | 0.007 | <0.0001 | 16.62 | 0.0008 |  | 0.001 | | 0.008 | | 0.9325 | | 5.04 | 0.1690 | | |
| rs13356951 (vs. CC) | -0.050 | 0.007 | <0.0001 | 16.29 | 0.0010 |  | 0.001 | | 0.008 | | 0.9068 | | 5.08 | 0.1662 | | |
| rs999428 (vs. CC) | -0.052 | 0.007 | <0.0001 | 16.28 | 0.0010 |  | 0.001 | | 0.008 | | 0.9085 | | 5.08 | 0.1658 | | |
| rs999427 (vs. AA) | -0.050 | 0.007 | <0.0001 | 15.84 | 0.0012 |  | 0.001 | | 0.008 | | 0.9600 | | 4.66 | 0.1987 | | |
| rs1504047 (vs. TT) | -0.050 | 0.007 | <0.0001 | 16.29 | 0.0008 |  | 0.001 | | 0.008 | | 0.8744 | | 5.43 | 0.1430 | | |
| rs11953193 (vs. CC) | -0.050 | 0.007 | <0.0001 | 15.51 | 0.0014 |  | -0.001 | | 0.008 | | 0.9799 | | 4.56 | 0.2072 | | |
| rs6870971 (vs. TT) | -0.052 | 0.007 | <0.0001 | 16.40 | 0.0009 |  | -0.001 | | 0.008 | | 0.9919 | | 5.14 | 0.1620 | | |
| rs10036293 (vs. GG) | -0.049 | 0.007 | <0.0001 | 15.02 | 0.0018 |  | 0.001 | | 0.008 | | 0.9096 | | 5.49 | 0.1391 | | |
| rs1536041 (vs. AA) | -0.050 | 0.007 | <0.0001 | 16.49 | 0.0009 |  | 0.001 | | 0.008 | | 0.8712 | | 5.16 | 0.1603 | | |
| rs2894536 (vs. GG) | -0.050 | 0.007 | <0.0001 | 16.56 | 0.0009 |  | 0.001 | | 0.008 | | 0.9921 | | 5.06 | 0.1675 | | |
| rs6977000 (vs. TT) | -0.050 | 0.007 | <0.0001 | 16.53 | 0.0009 |  | 0.001 | | 0.008 | | 0.9838 | | 5.02 | 0.1702 | | |
| rs10972486 (vs. TT) | -0.051 | 0.007 | <0.0001 | 16.69 | 0.0008 |  | -0.001 | | 0.008 | | 0.9043 | | 4.97 | 0.1737 | | |
| rs7898120 (vs. TT) | -0.049 | 0.007 | <0.0001 | 16.38 | 0.0009 |  | -0.001 | | 0.008 | | 0.9979 | | 5.00 | 0.1718 | | |
| rs16936709 (vs. CC) | -0.050 | 0.007 | <0.0001 | 16.55 | 0.0009 |  | 0.001 | | 0.008 | | 0.9875 | | 4.93 | 0.1773 | | |
| rs10734089 (vs. GG) | -0.049 | 0.006 | <0.0001 | 16.51 | 0.0009 |  | 0.001 | | 0.008 | | 0.9577 | | 4.97 | 0.1742 | | |
| rs1955011 (vs. TT) | -0.048 | 0.007 | <0.0001 | 15.98 | 0.0011 |  | 0.001 | | 0.008 | | 0.9080 | | 5.13 | 0.1626 | | |
| rs645809 (vs. AA) | -0.048 | 0.007 | <0.0001 | 16.53 | 0.0009 |  | 0.001 | | 0.008 | | 0.9838 | | 5.13 | 0.1626 | | |
| rs896993 (vs. CC) | -0.048 | 0.007 | <0.0001 | 14.41 | 0.0024 |  | 0.001 | | 0.008 | | 0.8682 | | 4.68 | 0.1972 | | |
| rs2373947 (vs. CC) | -0.049 | 0.007 | <0.0001 | 16.67 | 0.0008 |  | 0.001 | | 0.008 | | 0.9139 | | 4.98 | 0.1734 | | |
| rs16924259 (vs. AA) | -0.049 | 0.007 | <0.0001 | 16.01 | 0.0011 |  | 0.001 | | 0.008 | | 0.8757 | | 4.65 | 0.1991 | | |
| rs10501745 (vs. TT) | -0.051 | 0.007 | <0.0001 | 18.21 | 0.0004 |  | 0.001 | | 0.008 | | 0.8600 | | 4.90 | 0.1790 | | |
| rs6573780 (vs. AA) | -0.051 | 0.007 | <0.0001 | 16.45 | 0.0009 |  | -0.001 | | 0.008 | | 0.9519 | | 5.05 | 0.1679 | | |
| rs42923 (vs. CC) | -0.050 | 0.007 | <0.0001 | 16.61 | 0.0008 |  | 0.001 | | 0.008 | | 0.8801 | | 5.03 | 0.1694 | | |
| rs10220852 (vs. TT) | -0.051 | 0.007 | <0.0001 | 14.25 | 0.0026 |  | -0.001 | | 0.008 | | 0.8361 | | 5.83 | 0.1460 | | |
| rs17516321 (vs. TT) | -0.050 | 0.007 | <0.0001 | 16.64 | 0.0008 |  | -0.001 | | 0.008 | | 0.8989 | | 4.98 | 0.1735 | | |
| rs8070008 (vs. GG) | -0.051 | 0.007 | <0.0001 | 16.28 | 0.0010 |  | 0.001 | | 0.008 | | 0.9787 | | 5.26 | 0.1536 | | |
| rs8090956 (vs. CC) | -0.050 | 0.007 | <0.0001 | 16.42 | 0.0009 |  | 0.001 | | 0.008 | | 0.9115 | | 5.07 | 0.1670 | | |
| rs4798160 (vs. TT) | -0.048 | 0.007 | <0.0001 | 15.62 | 0.0014 |  | 0.001 | | 0.008 | | 0.4812 | | 4.90 | 0.1790 | | |
| rs2824571 (vs. CC) | -0.049 | 0.007 | <0.0001 | 16.47 | 0.0009 |  | 0.001 | | 0.008 | | 0.8512 | | 5.04 | 0.1691 | | |
| rs361594 (vs. TT) | -0.049 | 0.007 | <0.0001 | 16.48 | 0.0009 |  | 0.001 | | 0.008 | | 0.9608 | | 5.02 | 0.1706 | | |
| rs2894536 (vs. GA or AA) | -0.048 | 0.010 | <0.0001 | 16.46 | 0.0009 |  | 0.004 | | 0.009 | | <0.0001 | | 5.10 | 0.6701 | | |
| rs10972486 (vs. TC or CC) | -0.051 | 0.007 | <0.0001 | 16.65 | 0.0008 |  | 0.019 | | 0.008 | | 0.9181 | | 4.98 | 0.8229 | | |

Regression results were adjusted for age and sex.

SE, standard error.

^*^Estimated using the Durbin-Wu-Hausman test, which examines the difference between the estimates from ordinary least squares linear regression and instrumental variable analysis.

Supplementary Table S3. Multiple Cox proportional hazard model for hypertension (HTN)^*^ development

|  | Model I | |  | Model II | |  | Model III | |
| --- | --- | --- | --- | --- | --- | --- | --- | --- |
| Variable | HR | 95% *CI* |  | HR | 95% *CI* |  | HR | 95% *CI* |
| Systolic BP (mmHg) | 1.071 | 1.064-1.078 |  |  |  |  |  |  |
| Diastolic BP (mmHg) | 1.092 | 1.081-1.103 |  |  |  |  |  |  |
| Body mass index (kg/m^2^) | 1.071 | 1.048-1.095 |  |  |  |  |  |  |
| Waist circumference (cm) | 1.049 | 1.041-1.027 |  |  |  |  |  |  |
| White blood cell (10^9^/L) | 1.034 | 0.996-1.072 |  |  |  |  |  |  |
| Hemoglobin (g/dL) | 1.106 | 1.038-1.179 |  |  |  |  |  |  |
| Platelet (10^3^/μL) | 1.002 | 1.001-1.003 |  |  |  |  |  |  |
| Total protein (g/dL) | 0.883 | 0.756-1.031 |  |  |  |  |  |  |
| Albumin (g/dL) | 0.599 | 0.484-0.741 |  | 0.654 | 0.521-0.820 |  |  |  |
| Calcium (mg/dL) | 1.051 | 0.910-1.214 |  |  |  |  |  |  |
| Fasting glucose (mg/dL) | 1.004 | 0.996-1.012 |  |  |  |  |  |  |
| Post-prandial glucose (mg/dL) | 1.002 | 0.999-1.004 |  |  |  |  |  |  |
| Hemoglobin A1c (%) | 1.246 | 1.019-1.524 |  |  |  |  |  |  |
| eGFR^**^ (mL/min/1.73m^2^) | 0.997 | 0.991-1.002 |  |  |  |  |  |  |
| Total bilirubin (mg/dL) | 0.816 | 0.648-1.027 |  |  |  |  |  |  |
| Aspartate aminotransferase (IU/L) | 1.003 | 0.999-1.007 |  |  |  |  |  |  |
| Alanine aminotransferase (IU/L) | 1.003 | 1.001-1.005 |  |  |  |  |  |  |
| γ-Glutamyl transferase (IU/L) | 1.003 | 1.004-1.004 |  |  |  |  |  |  |
| Triglyceride (mg/dL) | 1.001 | 1.001-1.002 |  |  |  |  |  |  |
| HDL cholesterol (mg/dL) | 0.991 | 0.984-0.998 |  |  |  |  |  |  |
| LDL cholesterol (mg/dL) | 1.001 | 0.999-1.003 |  |  |  |  |  |  |
| C-reactive protein (mg/dL) | 1.047 | 0.912-1.202 |  |  |  |  |  |  |
| UACR (mg/g Cr) | 1.007 | 0.995-1.061 |  |  |  |  |  |  |
| rs3753613 |  |  |  |  |  |  |  |  |
| Additive model | 1.037 | 0.935-1.149 |  |  |  |  |  |  |
| Dominant model | 1.030 | 0.904-1.174 |  |  |  |  |  |  |
| Recessive model | 1.098 | 0.867-1.391 |  |  |  |  |  |  |
| rs2271933 |  |  |  |  |  |  |  |  |
| Additive model | 1.013 | 0.912-1.125 |  |  |  |  |  |  |
| Dominant model | 1.005 | 0.879-1.149 |  |  |  |  |  |  |
| Recessive model | 1.055 | 0.839-1.358 |  |  |  |  |  |  |
| rs4949454 |  |  |  |  |  |  |  |  |
| Additive model | 1.026 | 0.925-1.138 |  |  |  |  |  |  |
| Dominant model | 1.023 | 0.897-1.165 |  |  |  |  |  |  |
| Recessive model | 1.067 | 0.839-1.358 |  |  |  |  |  |  |
| rs3806368 |  |  |  |  |  |  |  |  |
| Additive model | 0.966 | 0.871-1.071 |  |  |  |  |  |  |
| Dominant model | 0.955 | 0.838-1.089 |  |  |  |  |  |  |
| Recessive model | 0.966 | 0.758-1.231 |  |  |  |  |  |  |
| rs1054943 |  |  |  |  |  |  |  |  |
| Additive model | 0.959 | 0.865-1.064 |  |  |  |  |  |  |
| Dominant model | 0.945 | 0.829-1.077 |  |  |  |  |  |  |
| Recessive model | 0.965 | 0.757-1.230 |  |  |  |  |  |  |
| rs9628673 |  |  |  |  |  |  |  |  |
| Additive model | 0.961 | 0.873-1.059 |  |  |  |  |  |  |
| Dominant model | 0.956 | 0.836-1.092 |  |  |  |  |  |  |
| Recessive model | 0.939 | 0.773-1.141 |  |  |  |  |  |  |
| rs2802809 |  |  |  |  |  |  |  |  |
| Additive model | 0.975 | 0.889-1.069 |  |  |  |  |  |  |
| Dominant model | 0.967 | 0.835-1.120 |  |  |  |  |  |  |
| Recessive model | 0.967 | 0.828-1.129 |  |  |  |  |  |  |
| rs13374930 |  |  |  |  |  |  |  |  |
| Additive model | 1.078 | 0.956-1.215 |  |  |  |  |  |  |
| Dominant model | 1.099 | 0.956-1.262 |  |  |  |  |  |  |
| Recessive model | 1.041 | 0.709-1.527 |  |  |  |  |  |  |
| rs780094 |  |  |  |  |  |  |  |  |
| Additive model | 1.065 | 0.970-1.168 |  |  |  |  |  |  |
| Dominant model | 1.014 | 0.878-1.171 |  |  |  |  |  |  |
| Recessive model | 1.180 | 1.010-1.378 |  | 1.177 | 1.009-1.373 |  | 1.158 | 0.991-1.353 |
| rs9860560 |  |  |  |  |  |  |  |  |
| Additive model | 0.976 | 0.887-1.074 |  |  |  |  |  |  |
| Dominant model | 0.963 | 0.844-1.099 |  |  |  |  |  |  |
| Recessive model | 0.980 | 0.794-1.169 |  |  |  |  |  |  |
| rs1504047 |  |  |  |  |  |  |  |  |
| Additive model | 0.972 | 0.883-1.069 |  |  |  |  |  |  |
| Dominant model | 0.963 | 0.844-1.099 |  |  |  |  |  |  |
| Recessive model | 0.963 | 0.794-1.169 |  |  |  |  |  |  |
| rs13356951 |  |  |  |  |  |  |  |  |
| Additive model | 0.990 | 0.870-1.126 |  |  |  |  |  |  |
| Dominant model | 0.998 | 0.863-1.153 |  |  |  |  |  |  |
| Recessive model | 0.909 | 0.577-1.433 |  |  |  |  |  |  |
| rs999428 |  |  |  |  |  |  |  |  |
| Additive model | 0.994 | 0.873-1.131 |  |  |  |  |  |  |
| Dominant model | 0.997 | 0.863-1.153 |  |  |  |  |  |  |
| Recessive model | 0.948 | 0.602-1.494 |  |  |  |  |  |  |
| rs999427 |  |  |  |  |  |  |  |  |
| Additive model | 0.972 | 0.853-1.108 |  |  |  |  |  |  |
| Dominant model | 0.974 | 0.840-1.129 |  |  |  |  |  |  |
| Recessive model | 0.920 | 0.584-1.450 |  |  |  |  |  |  |
| rs2963394 |  |  |  |  |  |  |  |  |
| Additive model | 1.003 | 0.881-1.141 |  |  |  |  |  |  |
| Dominant model | 1.008 | 0.872-1.164 |  |  |  |  |  |  |
| Recessive model | 0.958 | 0.608-1.796 |  |  |  |  |  |  |
| rs11953193 |  |  |  |  |  |  |  |  |
| Additive model | 1.116 | 0.973-1.280 |  |  |  |  |  |  |
| Dominant model | 1.138 | 0.974-1.329 |  |  |  |  |  |  |
| Recessive model | 1.111 | 0.687-1.796 |  |  |  |  |  |  |
| rs6870971 |  |  |  |  |  |  |  |  |
| Additive model | 0.988 | 0.898-1.086 |  |  |  |  |  |  |
| Dominant model | 1.000 | 0.875-1.143 |  |  |  |  |  |  |
| Recessive model | 0.952 | 0.787-1.152 |  |  |  |  |  |  |
| rs10036293 |  |  |  |  |  |  |  |  |
| Additive model | 0.977 | 0.898-1.086 |  |  |  |  |  |  |
| Dominant model | 0.996 | 0.872-1.138 |  |  |  |  |  |  |
| Recessive model | 0.914 | 0.748-1.117 |  |  |  |  |  |  |
| rs1536041 |  |  |  |  |  |  |  |  |
| Additive model | 0.887 | 0.771-1.020 |  |  |  |  |  |  |
| Dominant model | 0.889 | 0.760-1.040 |  |  |  |  |  |  |
| Recessive model | 0.701 | 0.413-1.189 |  |  |  |  |  |  |
| rs2894536 |  |  |  |  |  |  |  |  |
| Additive model | 1.164 | 1.022-1.325 |  | 1.158 | 1.017-1.319 |  | 1.141 | 1.003-1.299 |
| Dominant model | 1.188 | 1.026-1.376 |  | 1.185 | 1.024-1.371 |  | 1.176 | 1.015-1.361 |
| Recessive model | 1.225 | 0.777-1.931 |  |  |  |  |  |  |
| rs6977000 |  |  |  |  |  |  |  |  |
| Additive model | 1.087 | 0.965-1.225 |  |  |  |  |  |  |
| Dominant model | 1.107 | 0.964-1.271 |  |  |  |  |  |  |
| Recessive model | 1.078 | 0.745-1.561 |  |  |  |  |  |  |
| rs10972486 |  |  |  |  |  |  |  |  |
| Additive model | 1.090 | 0.990-1.201 |  |  |  |  |  |  |
| Dominant model | 1.160 | 1.015-1.325 |  | 1.153 | 1.008-1.317 |  | 1.152 | 1.009-1.316 |
| Recessive model | 1.047 | 0.854-1.282 |  |  |  |  |  |  |
| rs7898120 |  |  |  |  |  |  |  |  |
| Additive model | 1.041 | 0.933-1.160 |  |  |  |  |  |  |
| Dominant model | 1.030 | 0.902-1.177 |  |  |  |  |  |  |
| Recessive model | 1.143 | 0.868-1.505 |  |  |  |  |  |  |
| rs16936709 |  |  |  |  |  |  |  |  |
| Additive model | 1.017 | 0.864-1.196 |  |  |  |  |  |  |
| Dominant model | 0.995 | 0.834-1.186 |  |  |  |  |  |  |
| Recessive model | 1.450 | 0.777-2.704 |  |  |  |  |  |  |
| rs10734089 |  |  |  |  |  |  |  |  |
| Additive model | 1.012 | 0.879-1.165 |  |  |  |  |  |  |
| Dominant model | 1.005 | 0.900-1.226 |  |  |  |  |  |  |
| Recessive model | 0.618 | 0.320-1.191 |  |  |  |  |  |  |
| rs1955011 |  |  |  |  |  |  |  |  |
| Additive model | 0.993 | 0.898-1.097 |  |  |  |  |  |  |
| Dominant model | 1.013 | 0.889-1.154 |  |  |  |  |  |  |
| Recessive model | 0.928 | 0.739-1.167 |  |  |  |  |  |  |
| rs645809 |  |  |  |  |  |  |  |  |
| Additive model | 1.021 | 0.930-1.122 |  |  |  |  |  |  |
| Dominant model | 1.034 | 0.892-1.199 |  |  |  |  |  |  |
| Recessive model | 1.021 | 0.871-1.197 |  |  |  |  |  |  |
| rs896993 |  |  |  |  |  |  |  |  |
| Additive model | 1.031 | 0.885-1.201 |  |  |  |  |  |  |
| Dominant model | 1.028 | 0.874-1.209 |  |  |  |  |  |  |
| Recessive model | 1.158 | 0.577-2.326 |  |  |  |  |  |  |
| rs2373947 |  |  |  |  |  |  |  |  |
| Additive model | 1.004 | 0.852-1.183 |  |  |  |  |  |  |
| Dominant model | 0.989 | 0.832-1.177 |  |  |  |  |  |  |
| Recessive model | 1.383 | 0.657-2.913 |  |  |  |  |  |  |
| rs16924259 |  |  |  |  |  |  |  |  |
| Additive model | 0.952 | 0.864-1.701 |  |  |  |  |  |  |
| Dominant model | 0.948 | 0.827-1.088 |  |  |  |  |  |  |
| Recessive model | 0.910 | 0.639-1.295 |  |  |  |  |  |  |
| rs10501745 |  |  |  |  |  |  |  |  |
| Additive model | 0.918 | 0.815-1.035 |  |  |  |  |  |  |
| Dominant model | 0.913 | 0.793-1.051 |  |  |  |  |  |  |
| Recessive model | 0.841 | 0.588-1.204 |  |  |  |  |  |  |
| rs6573780 |  |  |  |  |  |  |  |  |
| Additive model | 1.023 | 0.933-1.121 |  |  |  |  |  |  |
| Dominant model | 1.004 | 0.867-1.163 |  |  |  |  |  |  |
| Recessive model | 1.059 | 0.909-1.234 |  |  |  |  |  |  |
| rs42923 |  |  |  |  |  |  |  |  |
| Additive model | 0.939 | 0.810-1.088 |  |  |  |  |  |  |
| Dominant model | 0.902 | 0.764-1.064 |  |  |  |  |  |  |
| Recessive model | 1.245 | 0.780-1.986 |  |  |  |  |  |  |
| rs10220852 |  |  |  |  |  |  |  |  |
| Additive model | 1.070 | 0.969-1.181 |  |  |  |  |  |  |
| Dominant model | 1.085 | 0.950-1.239 |  |  |  |  |  |  |
| Recessive model | 1.109 | 0.896-1.373 |  |  |  |  |  |  |
| rs17516321 |  |  |  |  |  |  |  |  |
| Additive model | 1.090 | 0.984-1.207 |  |  |  |  |  |  |
| Dominant model | 1.093 | 0.959-1.246 |  |  |  |  |  |  |
| Recessive model | 1.182 | 0.937-1.492 |  |  |  |  |  |  |
| rs8070008 |  |  |  |  |  |  |  |  |
| Additive model | 0.991 | 0.898-1.093 |  |  |  |  |  |  |
| Dominant model | 0.989 | 0.867-1.129 |  |  |  |  |  |  |
| Recessive model | 0.986 | 0.804-1.210 |  |  |  |  |  |  |
| rs8090956 |  |  |  |  |  |  |  |  |
| Additive model | 1.010 | 0.919-1.109 |  |  |  |  |  |  |
| Dominant model | 1.010 | 0.868-1.175 |  |  |  |  |  |  |
| Recessive model | 1.016 | 0.872-1.184 |  |  |  |  |  |  |
| rs4798160 |  |  |  |  |  |  |  |  |
| Additive model | 0.989 | 0.897-1.091 |  |  |  |  |  |  |
| Dominant model | 1.008 | 0.879-1.155 |  |  |  |  |  |  |
| Recessive model | 0.945 | 0.776-1.150 |  |  |  |  |  |  |
| rs2824571 |  |  |  |  |  |  |  |  |
| Additive model | 1.032 | 0.941-1.132 |  |  |  |  |  |  |
| Dominant model | 1.056 | 0.914-1.221 |  |  |  |  |  |  |
| Recessive model | 1.027 | 0.876-1.203 |  |  |  |  |  |  |
| rs361594 |  |  |  |  |  |  |  |  |
| Additive model | 0.992 | 0.904-1.090 |  |  |  |  |  |  |
| Dominant model | 0.964 | 0.833-1.114 |  |  |  |  |  |  |
| Recessive model | 1.023 | 0.873-1.198 |  |  |  |  |  |  |

^*^Defined as BP ≥ 140/90 mmHg and/or antihypertensive drug therapy during the follow-up period.

^**^Estimated using the Chronic Kidney Disease Epidemiology Collaboration (CKD-EPI) equation.

Model I: adjusted for age, sex, and smoking history.

Model II: adjusted for age, sex, smoking history, systolic BP, diastolic BP, body mass index, waist circumference, hemoglobin, platelet, hemoglobin A1c, alanine aminotransferase, γ-glutamyl transferase, triglyceride, and HDL-cholesterol levels.

Model III: adjusted for age, sex, smoking history, systolic BP, diastolic BP, body mass index, waist circumference, hemoglobin, platelet, albumin, hemoglobin A1c, alanine aminotransferase, γ-glutamyl transferase, triglyceride, and HDL-cholesterol levels.

HR, hazard ratio; *CI*, confidence interval
